# Hypergraph regularized nonnegative triple decomposition for multiway data analysis

**DOI:** 10.1038/s41598-024-59300-3

**Published:** 2024-04-20

**Authors:** Qingshui Liao, Qilong Liu, Fatimah Abdul Razak

**Affiliations:** 1 Department of Mathematical Sciences, Faculty of Science & Technology, Universiti Kebangsaan Malaysia, 43600 Bangi, Selangor Malaysia; 2 School of Mathematical Sciences, Guizhou Normal University, Guiyang, 550025 People’s Republic of China

**Keywords:** Nonnegative tensor decomposition, Triple decomposition, Hypergraph regularization, Data anaylsis, Computational science, Applied mathematics

## Abstract

Tucker decomposition is widely used for image representation, data reconstruction, and machine learning tasks, but the calculation cost for updating the Tucker core is high. Bilevel form of triple decomposition (TriD) overcomes this issue by decomposing the Tucker core into three low-dimensional third-order factor tensors and plays an important role in the dimension reduction of data representation. TriD, on the other hand, is incapable of precisely encoding similarity relationships for tensor data with a complex manifold structure. To address this shortcoming, we take advantage of hypergraph learning and propose a novel hypergraph regularized nonnegative triple decomposition for multiway data analysis that employs the hypergraph to model the complex relationships among the raw data. Furthermore, we develop a multiplicative update algorithm to solve our optimization problem and theoretically prove its convergence. Finally, we perform extensive numerical tests on six real-world datasets, and the results show that our proposed algorithm outperforms some state-of-the-art methods.

## Introduction

A massive amount of high-dimensional data has been accumulated in social networks, neural networks, data mining, computer vision, and other domains as data extraction technology has advanced. A number of issues arise when analyzing and processing high-dimensional data, such as the need for long computation times and large memory spaces. As a result, dimensionality reduction is commonly conducted prior to further processing and analysis of these data. High-dimensional data is often vectorized to form a larger matrix. Matrix-based methods, such as principal component analysis (PCA)^1^, singular value decomposition (SVD)^2^, multiway extensions of the SVD^3^, and linear discriminant analysis (LDA)^4^, are then used for dimensionality reduction. However, the matrix-based dimensionality reduction methods ignore the internal structure of the data. Therefore, tensor decomposition techniques are used to gain a better understanding of data features. There are some widely used tensor decomposition methods, such as Eckart-Young decomposition^5^, CANDECOMP/PARAFAC (CP) decomposition^6^, Tucker decomposition (TD)^7^, and the family of principal component decomposition models related to TD^8–12^. TD is the decomposition of a tensor into the product of the core tensor and some factor matrices in different directions. When the core tensor in TD is taken to be the unit tensor, it degenerates to CP decomposition. Different from CP, multiway versions of principal component decompositions related to TD focus on underlining different numbers of main influence components for various multiway data via feature extraction along different modes of models.

TD has been successfully applied in the fields of pattern recognition, cluster analysis, image denoising, and image complementation. Due to the powerful data representation capabilities of TD, many TD variants have been developed in recent years based on reasonable assumptions such as sparsity^13^, smoothness^14^, and convolution^15^. However, TD faces some challenges when dealing with high-dimensional data: (i) The size of the core tensor in TD grows rapidly as the order of the data increases, which may result in a high cost of calculation and estimation complexity; (ii) TD does not consider the variability in each direction. This variability is widespread in some real data, such as traffic and internet data, where the three modes of the third-order tensor have strong temporal, spatial, and periodic significance^16^. To remedy these shortcomings, Qi et al.^17^ proposed a bilevel form of triple decomposition (TriD). The triple decomposition for third-order tensors transforms a third-order tensor into a product of three third-order factor tensors. Each factor tensor represents a different meaning and is of lower dimension in two directions. TriD performs TD on a tensor and triple decomposes the Tucker core at the same time. The number of parameters in TriD is less than that of TD in substantial cases. Therefore, TriD is less costly than TD.

Although TriD has achieved better results in tensor data recovery experiments, it does not take into account the geometrical manifold structure of the data. In the past decade, manifold learning has been widely adopted to preserve the geometric information of original data. Cai et al.^18^ explored the geometrical information by constructing a *k*-nearest neighbor graph and proposed the graph regularized nonnegative matrix factorization (GNMF), which demonstrated promising performance in clustering analysis. To improve the robustness of GNMF, some variants of GNMF have been proposed as described in the literatiures^19–24^. Li et al.^25^ introduced a manifold regularization term on the core tensor and proposed a manifold regularization nonnegative Tucker decomposition (MR-NTD) method. Qiu et al.^26^ proposed a graph regularized nonnegaitve Tucker decomposition (GNTD) method by applying Laplacian regularization to the last nonnegative factor matrix. Liu et al.^27^ presented a technique known as graph regularized $L_p$ smooth NTD (GSNTD) via embedding graph regularization and $L_p$ smooth constraint into the original model of NTD. Subsequently, Wu et al.^28^ proposed a manifold regularization nonnegative triple decomposition (MRNTriD) of tensor sets that takes advantage of tensor geometry information. These graph-based manifold learning methods perform well in clustering. They, however, only consider the pairwise relationship between samples and ignore the high-order relationship among samples. Hypergraph learning is a good candidate for solving this problem.

Using a hypergraph to model the high-order relationship between samples will improve classification performance. There are numerous significant methods combined with hypergraphs that work well in clustering tasks: Zeng et al.^29^ presented a hypergraph regularized nonnegative matrix factorization (HNMF) method. Wang et al.^30^ introduced a hypergraph regularization to $L_{1/2}$-NMF (HSNMF) for exploiting spectral-spatial joint structure of hypespectral images. Huang et al.^31^ constructed a sparse hypergraph for better clustering and proposed a sparse hypergraph regularized NMF (SHNMF) method. Yin et al.^32^ proposed a hypergraph regularized nonnegative tensor factorization (HyperNTF) method by incorporating hypergraph into nonnegative tensor decomposition. Zhao et al.^33^ introduced a hypergraph regularized term into the framework of the nonnegative tensor ring decomposition and proposed a hypergraph regularized nonnegative tensor ring decomposition (HGNTR). To reduce computational complexity and suppress noise, they applied the low-rank approximation trick to accelerate HGNTR (LraHGNTR)^33^. Huang et al.^34^ designed a method to dynamically update the hypergraph and proposed a dynamic hypergraph regularized nonnegative Tucker decomposition (DHNTD) method.

To the best of our knowledge, there is no method to consider higher-order relationships among data sample points in TriD. Inspired by the advantages of hypergraph learning and TriD, in this paper, we present a hypergraph regularized nonnegative triple decomposition (HNTriD) model. HNTriD can explore low-dimensional parts-based representations while preserving detailed complex geometrical information from high-dimensional tensor data. Then, we develop an iterative multiplicative updating algorithm to solve the HNTriD model. The following are the main contributions of this paper:

- HNTriD is a novel dimensionality reduction method by incorporating hypergraph learning into TriD. It is good at dealing with the clustering tasks for tensor data, and the computation cost and containment resources could be greatly reduced.
- HNTriD embraces the merit of the complex connections of observed samples while retaining raw data structural information in dimensionality reduction. We attribute this excellent performance to the hypergraph regularized term’s ability which can successfully approximate the inner relationships of original data.
- HNTriD makes sense for some practical applications, such as clustering tasks, because it performs well at multiway data learning and can successfully preserve the important characteristics in dimensionality reduction. Experimental results in some popular datasets, including COIL20, GEORGIA, MNIST, ORL, PIE, and USPS, show that HNTriD outperforms existing rival approaches in cluster analysis.

The remainder of this paper is organized as follows: Section 2 goes over some fundamental concepts, such as NTD, TriD, and hypergraph learning, that will be used in the subsequent sections. The objective function of the HNTriD model is proposed in Section 3, and we discuss the HNTriD optimization algorithm in detail, including the updating rules for the parameters of the model, the convergence analysis of the proposed method, and the computation complexity analysis of HNTriD. In Section 4, we present some experimental results that can be used to validate the efficacy and accuracy of our proposed method. The last section is the conclusion.

## Related work

In this section, we briefly overview some basic definitions, including NTD^32,34,35^, TriD^17,25^, hypergraph learning^36–38^. The notations used in this paper are listed in Table 1.

**Table 1:** List of the notations relevant to this paper.

Notaions	Descriptions	Notations	Descriptions
$\textbf{U}$	A matrix	$\mathcal {X}$	A tensor
$\Vert \cdot \Vert _F$	Frobenius norm	$\text {Tr}(\cdot )$	Trace
$\llbracket \cdot \rrbracket$	Triple product	$\textbf{Y}_{(n)}$	The mode-*n* matricization of tensor $\mathcal {Y}$
$\otimes$	Kronecker product	$\mathcal {X}_{ijl}$	The (*i*, *j*, *l*) element of a third-order tensor
$\circledast$	Hadamard product	$\textrm{vec}(\cdot )$	The operator vectorizes a subject into a vector
$\mathcal {O}(\cdot )$	Computation cost	$\times _n$	The mode-*n* product

### Nonnegative tensor decomposition (NTD)

TD is a popular class of methods for dimensionality reduction of high-dimensional data^7^. The data collected in real life are usually nonnegative, so it makes more physical sense to add nonnegative constraints to all factors in TD. Therefore, we focus on the nonnegative tensor decomposition (NTD). In fact, NTD is a multiway extension of nonnegative matrix factorization (NMF)^39^, which imposes nonnegative constraints to the TD model^35^, and it preserves the multilinear structure of data. Given a nonnegative third-order tensor $\mathcal {X}\in \mathbb {R}_+^{n_1\times n_2\times n_3}$, NTD can be expressed as a core nonnegative tensor $\hat{\mathcal {X}}\in \mathbb {R}_+^{r_1\times r_2\times r_3}$ multiplied by three nonnegative factor matrices $\textbf{U}\in \mathbb {R}_+^{n_1\times r_1}$, $\textbf{V}\in \mathbb {R}_+^{n_2\times r_2}$, and $\textbf{W}\in \mathbb {R}_+^{n_3\times r_3}$, and it can be formulated as

1 $$\begin{aligned} \mathcal {X}=\hat{\mathcal {X}}\times _1\textbf{U}\times _2\textbf{V}\times _3\textbf{W}. \end{aligned}$$

If the smallest integers $r_1, r_2, r_3$ such that (1) holds, then we call the vector $(r_1, r_2, r_3)$ the Tucker rank. In the process of solving the optimal solution, we usually use its transformation of the mode-*n* matricization, and (1) can be expressed in the following equivalent forms

2 $$\begin{aligned} \textbf{X}_{(1)}=\textbf{U}\hat{\textbf{X}}_{(1)}\big (\textbf{W}\otimes \textbf{V}\big )^{\top }, \textbf{X}_{(2)}=\textbf{V}\hat{\textbf{X}}_{(2)}\big (\textbf{W}\otimes \textbf{U}\big )^{\top }, \text {and}\, \textbf{X}_{(3)}=\textbf{W}\hat{\textbf{X}}_{(3)}\big (\textbf{V}\otimes \textbf{U}\big )^{\top }, \end{aligned}$$

where $\textbf{X}_{(n)}$ denotes the mode-*n* matricization of the tensor $\mathcal {X}$, “$\otimes$” denotes the Kronecker product of two matrices.

### Bilevel form of triple decomposition (TriD)

In the TD and NTD methods, the size of the core tensor grows rapidly as the order of data increases, which may result in a high cost of calculation. To overcome this shortcoming, Qi et al.^17^ recently proposed a new form of triple decomposition for third-order tensors, which reduces a third-order tensor to the product of three third-order factor tensors.

#### Definition 1

^17^ Let $\hat{\mathcal {X}}=(\hat{x}_{ijl})\in \mathbb {R}^{r_1\times r_2\times r_3}$ be a nonzero tensor. We say that $\hat{\mathcal {X}}$ is the triple product of three third-order square tensors $\mathcal {A}\in \mathbb {R}^{r_1\times r\times r}$, $\mathcal {B}\in \mathbb {R}^{r\times r_2 \times r}$, and $\mathcal {C}\in \mathbb {R}^{r\times r\times r_3}$, triple product of the tensors is denoted by

3 $$\begin{aligned} \hat{\mathcal {X}}=\llbracket {\mathcal {A}\mathcal {B}\mathcal {C}}\rrbracket , \end{aligned}$$

where $\mathcal {A}$, $\mathcal {B}$, and $\mathcal {C}$ are named horizontally square tensor, laterally square tensor, and frontally square tensor, respectively. For $i=1,2,\ldots ,r_1, j=1,2,\ldots ,r_2$, $l=1,2,\ldots ,r_3$, the elementwise definition of the triple product can be illustrated as

4 $$\begin{aligned} \hat{\mathcal {X}}_{ijl}=\sum \limits _{p=1}^r \sum \limits _{q=1}^r \sum \limits _{s=1}^r \mathcal {A}_{iqs}\mathcal {B}_{pjs}\mathcal {C}_{pql}. \end{aligned}$$

If

$$\begin{aligned} r\le mid\{r_1,r_2,r_3\}, \end{aligned}$$

where “$mid\{\cdot \}$” denotes the median, we call (3) is a low rank triple decomposition of $\hat{\mathcal {X}}$. $\mathcal {A}, \mathcal {B}$, and $\mathcal {C}$ are the factor tensors of $\hat{\mathcal {X}}$. The smallest value of *r* such that (4) holds is known as the triple rank of $\hat{\mathcal {X}}$, which is denoted as TriRank($\hat{\mathcal {X}}$)=r. The triple rank of a zero tensor is defined as zero.

If a third-order tensor is decomposed by TD, and its Tucker core is triple decomposed into three tensors simultaneously. Then we get a bilevel form of the triple decomposition, that is shown below.

#### Definition 2

^17^ Based on the definition of NTD shown in (1), if the core tensor $\hat{\mathcal {X}}$ has a triple decomposition $\hat{\mathcal {X}}=\llbracket {\mathcal {A}\mathcal {B}\mathcal {C}}\rrbracket$, where TriRank($\hat{\mathcal {X}}$)=r, $\mathcal {A}\in \mathbb {R}_{+}^{r_1\times r\times r}$, $\mathcal {B}\in \mathbb {R}_{+}^{r\times r_2 \times r}$, and $\mathcal {C}\in \mathbb {R}_{+}^{r\times r \times r_3}$. Then $\mathcal {X}$ can be represented as

5 $$\begin{aligned} \mathcal {X}=\llbracket {\mathcal {A}\mathcal {B}\mathcal {C}}\rrbracket \times _1 \textbf{U}\times _2 \textbf{V}\times _3 \textbf{W}. \end{aligned}$$

We call (5) a bilevel form of the triple decomposition of $\mathcal {X}$, which is always referred as TriD. $\mathcal {A}$, $\mathcal {B}$, and $\mathcal {C}$ are the inner factor tensors.

From (1), the minimum number in parameters of NTD of the third-order tensor $\mathcal {X}$ is $n_1r_1+n_2r_2+n_3r_3+r_1r_2r_3$, where $(r_1, r_2, r_3)$ is the Tucker rank of $\mathcal {X}$. On the other hand, the number of parameters of TriD is $n_1r_1+n_2r_2+n_3r_3+(r_1+r_2+ r_3)r^2$, where *r* is the triple rank of $\hat{\mathcal {X}}$. Generally, the triple rank of $\hat{\mathcal {X}}$ is far less than each of the Tucker rank’s components of the original tensor $\mathcal {X}$. Then there are substantial cases where the number of parameters of TriD is strictly less than that of the TD.

NTD and TriD are linear dimensionality reduction techniques that may miss the essential nonlinear data structure. Manifold learning, on the other hand, is an effective technique for discovering geometric structure in multiway data, and hypergraph learning is a promising manifold learning method.

### Hypergraph learning

To improve clustering performance, it is necessary to maintain the internal hidden geometry structure information, which can be detailed by the hypergraph learning. Given $n_3$ grayscale image $\{\textbf{X}_1,\textbf{X}_2,\ldots , \textbf{X}_{n_3}\}$, each grayscale image can be viewed as a matrix of size $n_1\times n_2$. These $n_3$ matrices are stacked to form a tensor $\mathcal {X}$ of size $n_1\times n_2 \times n_3$. The *i*-th frontal slice $\mathcal {X}(:,:,i)$ of the tensor $\mathcal {X}$ is exactly the matrix $\textbf{X}_i$. In addition, we can build a hypergraph $(\mathbb {V},\mathbb {E};S)$ to encode the geometrical structure of raw data^40^. Each node $v_i\in \mathbb {V}$ represents a related data $\textbf{X}_i$ and every hyperedge $e_{i}\in \mathbb {E}$ consists of several nodes that are clustered by some constraints. For each vertex $v_i$, we form a hyperedge $e_i$ of $v_i$ and the *k*-neighbours of $v_i$. For each hyperedge $e_i$ with a weight $s(e_i)$ which is used to measure the similarity of the contained image nodes. The weight $s(e_i)$ can be calculated as follows:

6 $$\begin{aligned} s(e_i)=\sum _{\textbf{X}_j\in e_i}exp\bigg (\frac{-\Vert \textbf{X}_i-\textbf{X}_j\Vert _F^2}{\sigma ^2}\bigg ), \end{aligned}$$

where $\sigma =\frac{1}{k n_3}\sum _{i=1}^{n_3}\sum _{j\in e_i}\Vert \textbf{X}_i-\textbf{X}_j\Vert _F$ denotes the mean distance among all vertices in hyperedge $e_i$. In particular, we can construct an incidence matrix $\textbf{H}$ as follows:

$$\begin{aligned} \textbf{H}(v_i,e_q)=\left\{ \begin{array}{cc} 1, &{}\text {if}\quad v_i\in e_q,\\ 0, &{}\text {if}\quad v_i\notin e_q. \end{array} \right. \end{aligned}$$

The degrees of a node $v_i$ and a hyperedge $e_q$ can be expressed as

$$\begin{aligned} d(v_i)=\sum _{e_q\in \mathbb {E},v_i\in \mathbb {V}}s(e_q)\textbf{H}(v_i,e_q) \end{aligned}$$

and

$$\begin{aligned} d(e_q)=|e_q|=\sum _{v_i\in \mathbb {V}}\textbf{H}(v_i,e_q), \end{aligned}$$

respectively. We use $\textbf{D}_v, \textbf{D}_e$, and $\textbf{S}_e$ to denote diagonal matrices whose elements are $d(v_i)$, $d(e_q)$, and $s(e_q)$, respectively.

**Figure 1:**
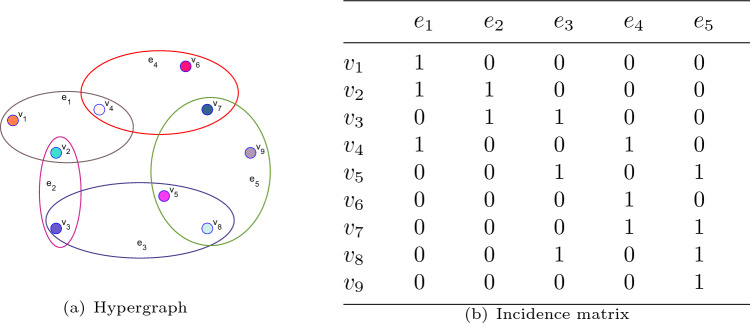
An example of hypergraph and its incident relationship.

To make the hypergraph more visual, we show the spatial structure in Figure 1. Herein every $v_j (j=1, 2, \dots , 9)$ represents a node, and each $e_i (i=1, 2, \dots , 5)$ denotes a hyperedge.

If two matrix data $\textbf{X}_i$ and $\textbf{X}_j$ are similar in the original raw observation, it is reasonable to assume that their low-dimensional representations $\textbf{w}_i$ and $\textbf{w}_j$ are adjacent to each other. Combined with practical application and theoretical analysis of hypergraph^31–33^, we can assume that $\textbf{w}_i$ and $\textbf{w}_j$ are the corresponding vectors that are related to the nodes $v_i$ and $v_j$. Then, the following expression can be used to calculate the clustering similarity of the original data $\textbf{X}_i$ and $\textbf{X}_j$ in the low-dimensional approximation.

$$\begin{aligned}{} & {} \frac{1}{2}\sum \limits _{e_q\in \mathbb {E}}\sum _{{v_i,v_j\in \mathbb {V}}}\dfrac{s(e_q)\textbf{H}(v_i,e_q)\textbf{H}(v_j,e_q)}{\delta (e_q)}\Vert \textbf{w}_i-\textbf{w}_j\Vert _2^2\\{} & {} \quad =\sum \limits _{e_q\in \mathbb {E}}\sum _{v_i\in \mathbb {V}}s(e_q)\textbf{H}(v_i,e_q)\Vert \textbf{w}_i\Vert _2^2\sum _{v_j\in \mathbb {V}}\dfrac{\textbf{H}(v_j,e_q)}{\delta (e_q)}\\{} & {} \qquad -\sum \limits _{e_q\in \mathbb {E}}\sum _{v_i,v_j\in \mathbb {V}}\dfrac{s(e_q)\textbf{H}(v_i,e_q)\textbf{H}(v_j,e_q)}{\delta (e_q)}{} \textbf{w}_i^{\top }{} \textbf{w}_j\\{} & {} \quad =\textrm{Tr}[\textbf{W}^\top (\textbf{D}_v-\textbf{H}\mathbf{{S}}_e\textbf{D}_{e} ^{-1}\textbf{H}^\top ) \textbf{W}]\\{} & {} \quad =\textrm{Tr}(\textbf{W}^\top \textbf{L}\textbf{W}), \end{aligned}$$

where $\textbf{L}=\textbf{D}_v-\bar{\textbf{S}}$ is a hypergraph Laplacian matrix that characterizes the data manifold, and $\bar{\textbf{S}}=\textbf{H}\mathbf{{S}}_e\textbf{D}_{e} ^{-1}\textbf{H}^\top$.

## Hypergraph regularized nonnegative triple decomposition (HNTriD)

TriD is a significant tensor data dimensional reduction algorithm, but it ignores higher-order relationships between the inner parts of raw data and does not consider nonnegative constraints, which may result in a big gap in data clustering performance. Modeling the high-order relationship among samples will help to improve performance. Hypergraph learning is an effective tool for illustrating the inner complex connections of multiway data. By incorporating the hypergraph Laplacian regularized term into the bilevel form of triple decomposition, we get a new method named HNTriD, as shown in the following subsection.

### Objective function of HNTriD

Suppose $\mathcal {X}\in \mathbb {R}_{+}^{n_1\times n_2 \times n_3}$ be a third-order nonnegative tensor which we stack the samples that are represented by $n_3$ second-order data $\textbf{X}_i\in \mathbb {R}_{+}^{n_1\times n_2} (i=1,2,\ldots ,n_3)$ as the elements of the third mode, each $\textbf{X}_i$ represents an original data sample of the raw data. Note that $\textbf{X}_{(3)}=[\textrm{vec}{(\textbf{X}_1)},\textrm{vec}{(\textbf{X}_2)},\ldots ,\textrm{vec}{(\textbf{X}_3)}]^\top$, unfolding $\mathcal {X}$ along the third mode we can simplify (1) into its matricization form that equals to the third equation of (2), which can be written as

$$\begin{aligned} \textbf{X}_{(3)}^\top =\left[ (\textbf{V}\otimes \textbf{U})\hat{\textbf{X}}_{(3)}^\top \right] \textbf{W}^\top . \end{aligned}$$

Let $\textbf{W}=[\textbf{w}_1,\textbf{w}_2,\ldots ,\textbf{w}_{n_3}]^\top$, each $\textbf{w}_k\in \mathbb {R}_+^{n_3}$ can be regarded as a low-dimensional representation for the data $\textbf{X}_k$ under the basis of $(\textbf{V}\otimes \textbf{U})\hat{\textbf{X}}_{(3)}^\top$.

To improve the multiway data representation ability and brush up operational efficiency, we propose the following HNTriD model, which incorporates the hypergraph constraint into the TriD model. For a given nonnegative tensor, $\mathcal {X}\in \mathbb {R}_{+}^{n_1\times n_2\times n_3}$, HNTriD aims to find three nonnegative tensors $\mathcal {A}\in \mathbb {R}_{+}^{r_1\times r\times r}$, $\mathcal {B}\in \mathbb {R}_{+}^{r\times r_2 \times r}$, and $\mathcal {C}\in \mathbb {R}_{+}^{r\times r \times r_3}$ and three nonnegative factor matrices $\textbf{U}\in \mathbb {R}_{+}^{n_1\times r_1}$, $\textbf{V}\in \mathbb {R}_{+}^{n_2\times r_2}$, and $\textbf{W}\in \mathbb {R}_{+}^{n_3\times r_3}$ such that

7 $$\begin{aligned}{} & {} \min \limits _{\mathcal {A},\mathcal {B},\mathcal {C},\textbf{U},\textbf{V},\textbf{W}} f_{HNTriD}=\frac{1}{2}\Vert \mathcal {X}-\llbracket {\mathcal {A}\mathcal {B}\mathcal {C}}\rrbracket \times _1\textbf{U}\times _2\textbf{V}\times _3\textbf{W}\Vert _F^2+\frac{\alpha }{2}\textrm{Tr}(\textbf{W}^\top \textbf{L}\textbf{W}),\nonumber \\{} & {} s.t. \mathcal {A}\ge 0, \mathcal {B}\ge 0, \mathcal {C}\ge 0, \textbf{U}\ge 0, \textbf{V}\ge 0, \textbf{W}\ge 0. \end{aligned}$$

The first and second parts of (7) are the reconstruction error term and the hypergraph regularized term, respectively. The reconstruction error term in (7) can be seen as a deep nonnegative tensor decomposition with two layers. The first layer is a TD in the following form

$$\begin{aligned}\mathcal {X}\approx \mathcal {Y}\times _1\textbf{U}\times _2\textbf{V}\times _3\textbf{W},\end{aligned}$$

where $\mathcal {Y}\times _1\textbf{U}\times _2\textbf{V}$ denotes the set of multilinear bases of the original data $\mathcal {X}$ and $\textbf{W}$ denotes the encoding matrix of $\mathcal {X}$ under this set of multilinear bases. The second layer is the triple decomposition, which takes the following form

$$\begin{aligned} \mathcal {Y}\approx \llbracket {\mathcal {A}\mathcal {B}\mathcal {C}}\rrbracket , \end{aligned}$$

where each factor tensor represents a different meaning in different application problems. For example, in social networks and transportation data, different characteristics such as temporal stability, spatial correlation, and traffic periodicity may be reflected in each of these three factors. This two-layer decomposition not only reduces the computation required to update the core tensor, but also takes into account the respective advantages of the TD and the triple decomposition. The variable $\alpha$ is an adjustment parameter that is used to measure the importance of the hypergraph regularization term. The hypergraph regularization term preserves the multilateral relationships among the data, so we establish model (7).

**Figure 2:**
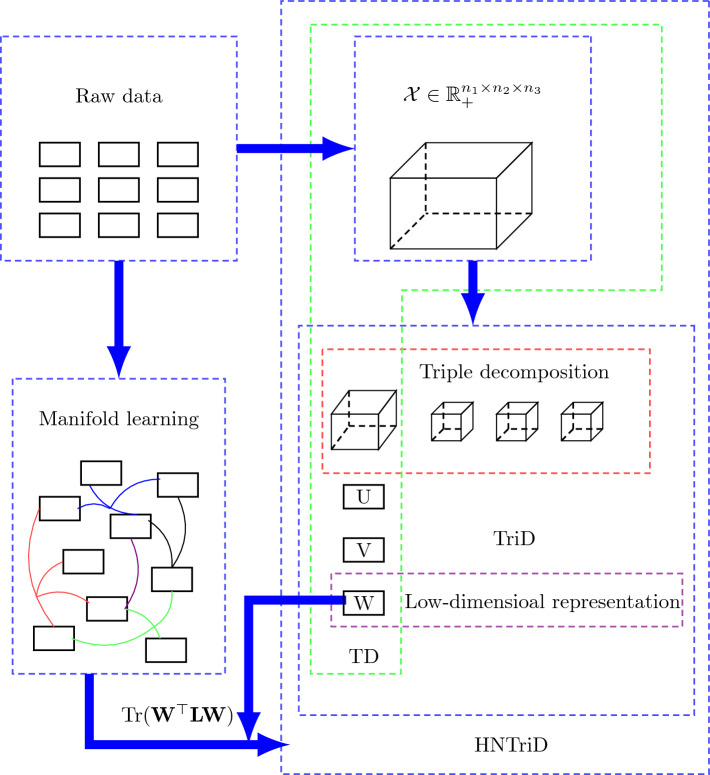
A flowchart used to show the implementation process of HNTriD in data analysis.

HNTriD is used to represent high-dimensional data in a low-dimensional form. To better show the implications of HNTriD, we draw a flowchart to provide a concise overview of the implementation procedure in Figure 2.

### Optimization algorithm

When the parameters $\mathcal {A}, \mathcal {B}, \mathcal {C}, \textbf{U}, \textbf{V}$, and $\textbf{W}$ are considered simultaneously, the objective function $f_{HNTriD}$ of HNTriD in (7) is not convex. Therefore, obtaining the global optimal solution is difficult. To deal with it, we introduce an iterative algorithm that achieves a local minimum. To simplify the process of solving the optimal algorithm, we show two important lemmas that will be frequently used.

#### Lemma 1

^17^Let $\hat{\mathcal {X}}=\llbracket {\mathcal {A}\mathcal {B}\mathcal {C}}\rrbracket$, we define three third-order tensors $\mathcal {F}\in \mathbb {R}_{+}^{r^2\times r_2\times r_3}$, $\mathcal {G}\in \mathbb {R}_{+}^{r_1\times r^2 \times r_3}$, and $\mathcal {H}\in \mathbb {R}_{+}^{r_1\times r_2\times r^2}$ with entries

8 $$\begin{aligned} \mathcal {F}_{kjt}=\sum \limits _{p=1}^r\mathcal {B}_{pjs}\mathcal {C}_{pqt},\quad \mathcal {G}_{ilt}=\sum \limits _{q=1}^r\mathcal {A}_{iqs}\mathcal {C}_{pqt},\quad \text {and} \quad \mathcal {H}_{ijm}=\sum \limits _{s=1}^r\mathcal {A}_{iqs}\mathcal {B}_{pjs}, \end{aligned}$$

where $k=q+(s-1)r$, $l=p+(s-1)r$, and $m=p+(q-1)r$, respectively. Then

$$\begin{aligned} \hat{\mathcal {X}}=\mathcal {F}\times _{1}\textbf{A}_{(1)}=\mathcal {G}\times _2 \textbf{B}_{(2)}=\mathcal {H}\times _3 \textbf{C}_{(3)}. \end{aligned}$$

#### Lemma 2

Let $\textbf{M}\in \mathbb {R}^{m\times n}$, $\textbf{N}\in \mathbb {R}^{n\times p}$, $\textbf{P}\in \mathbb {R}^{p\times q}$, and $\textbf{Q}\in \mathbb {R}^{m\times q}$. Then

9 $$\begin{aligned} \frac{\partial \Vert \textbf{Q}-\textbf{M}\textbf{N}\textbf{P}\Vert _F^2}{\partial \textbf{N}}=2 \textbf{M}^\top (\textbf{M}\textbf{N}\textbf{P}-\textbf{Q})\textbf{P}^\top . \end{aligned}$$

#### Proof

According to

$$\begin{aligned} \Vert \textbf{Q}-\textbf{M}\textbf{N}\textbf{P}\Vert _F^2= & {} \textrm{Tr}\left[ (\textbf{Q}-\textbf{M}\textbf{N}\textbf{P})^\top (\textbf{Q}-\textbf{M}\textbf{N}\textbf{P})\right] \\= & {} \textrm{Tr}\left[ (\textbf{M}\textbf{N}\textbf{P})^\top (\textbf{M}\textbf{N}\textbf{P})\right] -2\textrm{Tr}(\textbf{P}\textbf{Q}^\top \textbf{M}\textbf{N})+\textrm{Tr}(\textbf{Q}^\top \textbf{Q}), \end{aligned}$$

one has

$$\begin{aligned} \frac{\partial \Vert \textbf{Q}-\textbf{M}\textbf{N}\textbf{P}\Vert _F^2}{\partial \textrm{vec}{(\textbf{N})}}=\frac{\partial \textrm{Tr}\left[ (\textbf{M}\textbf{N}\textbf{P})^\top (\textbf{M}\textbf{N}\textbf{P})\right] }{\partial \textrm{vec}{(\textbf{N})}} -2\frac{\partial \textrm{Tr}(\textbf{P}\textbf{Q}^\top \textbf{M}\textbf{N})}{\partial \textrm{vec}{(\textbf{N})}}. \end{aligned}$$

Combining it with

$$\begin{aligned} \frac{\partial \textrm{Tr}\left[ (\textbf{M}\textbf{N}\textbf{P})^\top (\textbf{M}\textbf{N}\textbf{P})\right] }{\partial \textrm{vec}{(\textbf{N})}}= & {} \frac{\partial \textrm{Tr}\left[ (\textbf{M}\textbf{N}\textbf{P})^\top (\textbf{M}\textbf{N}\textbf{P})\right] }{\partial \textrm{vec}{(\textbf{M}\textbf{N}\textbf{P})}} \cdot \frac{\partial \textrm{vec}{(\textbf{M}\textbf{N}\textbf{P})}}{\partial \textrm{vec}{(\textbf{M}\textbf{N})}}\cdot \frac{\partial \textrm{vec}{(\textbf{M}\textbf{N})}}{\partial \textrm{vec}{(\textbf{N})}}\\= & {} 2\left[ \textrm{vec}(\textbf{M}\textbf{N}\textbf{P})\right] ^\top (\textbf{P}^\top \otimes \textbf{I}_m)(\textbf{I}_q \otimes \textbf{M})\\= & {} 2 \left[ \textrm{vec}(\textbf{M}\textbf{N}\textbf{P})\right] ^\top (\textbf{P}^\top \otimes \textbf{M})\\= & {} 2 \left[ \textrm{vec}(\textbf{M}^\top \textbf{M}\textbf{N}\textbf{P}\textbf{P}^\top )\right] ^\top \end{aligned}$$

and

$$\begin{aligned} \frac{\partial \textrm{Tr}(\textbf{P}\textbf{Q}^\top \textbf{M}\textbf{N})}{\partial \textrm{vec}{(\textbf{N})}}=\left[ \textrm{vec}(\textbf{M}^\top \textbf{Q}\textbf{P}^\top )\right] ^\top \end{aligned}$$

yields

$$\begin{aligned} \frac{\partial \Vert \textbf{Q}-\textbf{M}\textbf{N}\textbf{P}\Vert _F^2}{\partial \textrm{vec}{(\textbf{N})}}=2 \textrm{vec}\left[ \textbf{M}^\top (\textbf{M}\textbf{N}\textbf{P}-\textbf{Q})\textbf{P}^\top \right] ^\top . \end{aligned}$$

Therefore, $\frac{\partial \Vert \textbf{M}\textbf{N}\textbf{P}-\textbf{Q}\Vert _F^2}{\partial \textbf{N}}=2 \textbf{M}^\top (\textbf{M}\textbf{N}\textbf{P}-\textbf{Q})\textbf{P}^\top$ is obtained. This completes the proof. $\square$

#### Solutions of inner factor tensors

When the variables $\mathcal {B}, \mathcal {C}, \textbf{U}, \textbf{V}$, and $\textbf{W}$ are fixed, then the objective function of HNTriD is equivalent to

10 $$\begin{aligned} \min \limits _{\mathcal {A}\ge 0}\frac{1}{2}\Vert \mathcal {X}-\llbracket {\mathcal {A}\mathcal {B}\mathcal {C}}\rrbracket \times _1\textbf{U}\times _2\textbf{V}\times _3\textbf{W}\Vert _F^2. \end{aligned}$$

The Lagrange function of the above optimization problem (10) is

11 $$\begin{aligned} L_{\mathcal {A}}=\frac{1}{2}\Vert \mathcal {X}-\llbracket {\mathcal {A}\mathcal {B}\mathcal {C}}\rrbracket \times _1\textbf{U}\times _2\textbf{V}\times _3\textbf{W}\Vert _F^2-\textrm{Tr}(\Phi _1\textbf{A}_{(1)}^\top ). \end{aligned}$$

The matricization form of (11) that along the mode-1 is

$$\begin{aligned} L_{\mathcal {A}}=\frac{1}{2}\Vert \textbf{X}_{(1)}-\textbf{U}\textbf{A}_{(1)}\textbf{F}_{(1)}(\textbf{W}\otimes \textbf{V})^\top \Vert _F^2-\textrm{Tr}(\Phi _1\textbf{A}_{(1)}^\top ), \end{aligned}$$

where $\textbf{F}_{(1)}$ is the unfolding form of $\mathcal {F}$ that defined as (8). By Lemma 2, the gradient of $L_{\mathcal {A}}$ with respect to $\textbf{A}_{(1)}$ is given by

$$\begin{aligned} \frac{\partial L_{\mathcal {A}}}{\partial \textbf{A}_{(1)}}=\textbf{U}^\top \left( \textbf{U}\textbf{A}_{(1)}\textbf{F}_{(1)}(\textbf{W}\otimes \textbf{V})^\top -\textbf{X}_{(1)}\right) (\textbf{W}\otimes \textbf{V})\textbf{F}_{(1)}^\top -\Phi _1. \end{aligned}$$

According to^41^, we can take advantage of the Karush-Kuhn-Tucker (KKT) conditions $\frac{\partial L_{\mathcal {A}}}{\partial \textbf{A}_{(1)}}=\textbf{0}$ and $\Phi _1 \circledast \textbf{A}_{(1)}=\textbf{0}$, then the following equation is satisfied,

$$\begin{aligned}&\left( \textbf{U}^\top \textbf{U}\textbf{A}_{(1)}\textbf{F}_{(1)}(\textbf{W}\otimes \textbf{V})^\top (\textbf{W}\otimes \textbf{V})\textbf{F}_{(1)}^\top \right) \circledast \textbf{A}_{(1)} -\left( \textbf{U}^\top \textbf{X}_{(1)}(\textbf{W}\otimes \textbf{V})\textbf{F}_{(1)}^\top \right) \circledast \textbf{A}_{(1)}=\textbf{0}. \end{aligned}$$

Based on the above equation, we obtain the following updating rule for $\mathcal {A}$, and

12 $$\begin{aligned} {[}\textbf{A}_{(1)}]_{ij}\longleftarrow [\textbf{A}_{(1)}]_{ij}\frac{\left[ \textbf{U}^\top \textbf{X}_{(1)}(\textbf{W}\otimes \textbf{V})\textbf{F}_{(1)}^\top \right] _{ij}}{\left[ \textbf{U}^\top \textbf{U}\textbf{A}_{(1)}\textbf{F}_{(1)}(\textbf{W}\otimes \textbf{V})^\top (\textbf{W}\otimes \textbf{V})\textbf{F}_{(1)}^\top \right] _{ij}}. \end{aligned}$$

Using the same technique, updating rules for inner factor tensors $\mathcal {B}$ and $\mathcal {C}$ are obtained, which can be expressed as

13 $$\begin{aligned} {[}\textbf{B}_{(2)}]_{ij}\longleftarrow [\textbf{B}_{(2)}]_{ij} \frac{\left[ \textbf{V}^\top \textbf{X}_{(2)}(\textbf{W}\otimes \textbf{U})\textbf{G}_{(2)}^\top \right] _{ij}}{\left[ \textbf{V}^\top \textbf{V}\textbf{B}_{(2)}\textbf{G}_{(2)}(\textbf{W}\otimes \textbf{U})^\top (\textbf{W}\otimes \textbf{U})\textbf{G}_{(2)}^\top \right] _{ij}} \end{aligned}$$

and

14 $$\begin{aligned} {[}\textbf{C}_{(3)}]_{ij}\longleftarrow [\textbf{C}_{(3)}]_{ij} \frac{\left[ \textbf{W}^\top \textbf{X}_{(3)}(\textbf{V}\otimes \textbf{U})\textbf{H}_{(3)}^\top \right] _{ij}}{\left[ \textbf{W}^\top \textbf{W}\textbf{C}_{(3)}\textbf{H}_{(3)}(\textbf{V}\otimes \textbf{U})^\top (\textbf{V}\otimes \textbf{U})\textbf{H}_{(3)}^\top \right] _{ij}}, \end{aligned}$$

respectively.

#### Solutions of factor matrices

When the variables $\mathcal {A}, \mathcal {B}, \mathcal {C}, \textbf{U}$, and $\textbf{V}$ are fixed, then the objective function of HNTriD is equivalent to

15 $$\begin{aligned} \min \limits _{\textbf{W}\ge 0}\frac{1}{2}\Vert \mathcal {X}-\llbracket {\mathcal {A}\mathcal {B}\mathcal {C}}\rrbracket \times _1\textbf{U}\times _2\textbf{V}\times _3\textbf{W}\Vert _F^2+\frac{\alpha }{2}\textrm{Tr}(\textbf{W}^\top \textbf{L}\textbf{W}). \end{aligned}$$

The Lagrange function of the optimization problem (15) is

16 $$\begin{aligned} L_{\textbf{W}}=\frac{1}{2}\Vert \mathcal {X}-\llbracket {\mathcal {A}\mathcal {B}\mathcal {C}}\rrbracket \times _1\textbf{U}\times _2\textbf{V}\times _3\textbf{W}\Vert _F^2+\frac{\alpha }{2}\textrm{Tr}(\textbf{W}^\top \textbf{L}\textbf{W})-\textrm{Tr}(\Psi _3\textbf{W}^\top ). \end{aligned}$$

By using a transformation of the mode-3 matricization of the tensor $\mathcal {X}$ and $\hat{\mathcal {X}}$, (16) is obtained as follows

$$\begin{aligned} L_{\textbf{W}}=\frac{1}{2}\Vert \textbf{X}_{(3)}-\textbf{W}\textbf{C}_{(3)}\textbf{H}_{(3)}(\textbf{V}\otimes \textbf{U})^\top \Vert _F^2+\frac{\alpha }{2}\textrm{Tr}(\textbf{W}^\top \textbf{L}\textbf{W})-\textrm{Tr}(\Psi _3\textbf{W}^\top ). \end{aligned}$$

By Lemma 2, the gradient of $L_{\textbf{W}}$ with respect to $\textbf{W}$ is given by

$$\begin{aligned} \frac{\partial L_{\textbf{W}}}{\partial \textbf{W}}=\left( \textbf{W}\textbf{C}_{(3)}\textbf{H}_{(3)}(\textbf{V}\otimes \textbf{U})^\top -\textbf{X}_{(3)}\right) (\textbf{V}\otimes \textbf{U})\textbf{H}_{(3)}^\top \textbf{C}_{(3)}^\top +\alpha \textbf{L}\textbf{W}-\Psi _3. \end{aligned}$$

Using the Karush-Kuhn-Tucker (KKT) conditions $\frac{\partial L_{\textbf{W}}}{\partial \textbf{W}}=\textbf{0}$ and $\Psi _3 \circledast \textbf{W}=\textbf{0}$, the following equation is satisfied,

$$\begin{aligned}{} & {} \left( \textbf{W}\textbf{C}_{(3)}\textbf{H}_{(3)}(\textbf{V}\otimes \textbf{U})^\top (\textbf{V}\otimes \textbf{U})\textbf{H}_{(3)}^\top \textbf{C}_{(3)}^\top +\alpha \textbf{D}_{v} \textbf{W}\right) \circledast \textbf{W}\\{} & {} \quad -\left( \textbf{X}_{(3)}(\textbf{V}\otimes \textbf{U})\textbf{H}_{(3)}^\top \textbf{C}_{(3)}^\top +\alpha \bar{\textbf{S}} \textbf{W}\right) \circledast \textbf{W}=\textbf{0}. \end{aligned}$$

Based on the above equation, we obtain the following updating rule for $\textbf{W}$, and

17 $$\begin{aligned} \textbf{W}_{ij}\longleftarrow \textbf{W}_{ij}\frac{\left[ \textbf{X}_{(3)}(\textbf{V}\otimes \textbf{U})\textbf{H}_{(3)}^\top \textbf{C}_{(3)}^\top +\alpha \bar{\textbf{S}} \textbf{W}\right] _{ij}}{\left[ \textbf{W}\textbf{C}_{(3)}\textbf{H}_{(3)}(\textbf{V}\otimes \textbf{U})^\top (\textbf{V}\otimes \textbf{U})\textbf{H}_{(3)}^\top \textbf{C}_{(3)}^\top +\alpha \textbf{D}_{v} \textbf{W}\right] _{ij}}. \end{aligned}$$

Using the same technique, updating rules for the inner factor matrices $\textbf{U}$ and $\textbf{V}$ are obtained, which can presented as

18 $$\begin{aligned} \textbf{U}_{ij}\longleftarrow \textbf{U}_{ij}\frac{\left[ \textbf{X}_{(1)}(\textbf{W}\otimes \textbf{V})\textbf{F}_{(1)}^\top \textbf{A}_{(1)}^\top \right] _{ij}}{\left[ \textbf{U}\textbf{A}_{(1)}\textbf{F}_{(1)}(\textbf{W}\otimes \textbf{V})^\top (\textbf{W}\otimes \textbf{V})\textbf{F}_{(1)}^\top \textbf{A}_{(1)}^\top \right] _{ij}} \end{aligned}$$

and

19 $$\begin{aligned} \textbf{V}_{ij}\longleftarrow \textbf{V}_{ij} \frac{\left[ \textbf{X}_{(2)}(\textbf{W}\otimes \textbf{U})\textbf{G}_{(2)}^\top \textbf{B}_{(2)}^\top \right] _{ij}}{\left[ \textbf{V}\textbf{B}_{(2)}\textbf{G}_{(2)}(\textbf{W}\otimes \textbf{U})^\top (\textbf{W}\otimes \textbf{U})\textbf{G}_{(2)}^\top \textbf{B}_{(2)}^\top \right] _{ij}}, \end{aligned}$$

respectively.

### Convergence analysis theorically

In this subsection, the convergence of the iterative updating algorithm is investigated. Our proof will make use of an auxiliary function that is defined as below.

#### Definition 3

^42^ $\mathbb {G}(x,\tilde{x})$ is an auxiliary function for $\mathbb {F}(x)$ if the conditions

$$\begin{aligned} \mathbb {G}(x,\tilde{x})\ge \mathbb {F}(x) \quad \text {and} \quad \mathbb {G}(x,x)=\mathbb {F}(x) \end{aligned}$$

are satisfied.

The auxiliary function is of great help due to the key property that is shown as follows:

#### Lemma 3

^42^ If $\mathbb {G}(x,\tilde{x})$ is an auxiliary function of $\mathbb {F}(x)$, then $\mathbb {F}(x)$ is non-increasing under the update

20 $$\begin{aligned} x^{t+1}=\arg \min \limits _{x}\mathbb {G}(x,x^{t}). \end{aligned}$$

Now, we are going to show that the update rule for $\textbf{A}_{(1)}$ shown in (12) is exactly the same as that shown in (20) with a proper auxiliary function. Considering the *i*th row and *j*th column entry $[\textbf{A}_{(1)}]_{ij}$ in $\textbf{A}_{(1)}$, we use $\mathbb {F}_{ij}$ to denote the part of the objective function (7) that is relevant only to $[\textbf{A}_{(1)}]_{ij}$. The first and second derivatives of $\mathbb {F}_{ij}$ are

$$\begin{aligned} \mathbb {F}_{ij}^{\prime }=\left[ \textbf{U}^\top \left( \textbf{U}\textbf{A}_{(1)}\textbf{F}_{(1)}(\textbf{W}\otimes \textbf{V})^\top -\textbf{X}_{(1)}\right) (\textbf{W}\otimes \textbf{V})\textbf{F}_{(1)}^\top \right] _{ij} \end{aligned}$$

and

$$\begin{aligned} \mathbb {F}_{ij}^{\prime \prime }= (\textbf{U}^\top \textbf{U})_{ii}\left( \textbf{F}_{(1)}(\textbf{W}\otimes \textbf{V})^\top (\textbf{W}\otimes \textbf{V})\textbf{F}_{(1)}^\top \right) _{jj}, \end{aligned}$$

respectively.

#### Lemma 4

The function

21 $$\begin{aligned} \mathbb {G}(x,[\textbf{A}_{(1)}]_{ij}^t)= & {} \mathbb {F}_{ij}([\textbf{A}_{(1)}]_{ij}^t)+\mathbb {F}_{ij}^\prime ([\textbf{A}_{(1)}]_{ij}^t)(x-[\textbf{A}_{(1)}]_{ij}^t)\nonumber \\{} & {} +\frac{\left[ \textbf{U}^\top \textbf{U}\textbf{A}_{(1)}\textbf{F}_{(1)}(\textbf{W}\otimes \textbf{V})^\top (\textbf{W}\otimes \textbf{V})\textbf{F}_{(1)}^\top \right] _{ij}}{2[\textbf{A}_{(1)}]_{ij}^t}(x-[\textbf{A}_{(1)}]_{ij}^t)^2 \end{aligned}$$

is an auxiliary function for $\mathbb {F}_{ij}$, which is only relevant to $[\textbf{A}_{(1)}]_{ij}$.

#### Proof

Since $\mathbb {G}(x,x)=\mathbb {F}_{ij}(x)$ is obvious, we only need to show that the condition $\mathbb {G}(x,[\textbf{A}_{(1)}]_{ij}^t)\ge \mathbb {F}_{ij}(x)$ holds. To achieve this, we take into consideration the Taylor series expansion of $\mathbb {F}_{ij}(x)$ which can be formalized as

22 $$\begin{aligned} \mathbb {F}_{ij}(x)=\mathbb {F}_{ij}([\textbf{A}_{(1)}]_{ij}^t)+\mathbb {F}_{ij}^\prime ([\textbf{A}_{(1)}]_{ij}^t)(x-[\textbf{A}_{(1)}]_{ij}^t)+\frac{1}{2}\mathbb {F}_{ij}^{\prime \prime }([\textbf{A}_{(1)}]_{ij}^t)(x-[\textbf{A}_{(1)}]_{ij}^t)^2. \end{aligned}$$

Comparing (21) with (22), we can get that $\mathbb {G}(x,[\textbf{A}_{(1)}]_{ij}^t)\ge \mathbb {F}_{ij}(x)$ is satisfied as long as

$$\begin{aligned} \frac{\left[ \textbf{U}^\top \textbf{U}\textbf{A}_{(1)}\textbf{F}_{(1)}(\textbf{W}\otimes \textbf{V})^\top (\textbf{W}\otimes \textbf{V})\textbf{F}_{(1)}^\top \right] _{ij}}{2[\textbf{A}_{(1)}]_{ij}^t}\ge \frac{1}{2} \mathbb {F}_{ij}^{\prime \prime }([\textbf{A}_{(1)}]_{ij}^t) \end{aligned}$$

holds, which can be expressed as

23 $$\begin{aligned}{} & {} \left[ \textbf{U}^\top \textbf{U}\textbf{A}_{(1)}\textbf{F}_{(1)}(\textbf{W}\otimes \textbf{V})^\top (\textbf{W}\otimes \textbf{V})\textbf{F}_{(1)}^\top \right] _{ij}\nonumber \\{} & {} \quad \ge [\textbf{A}_{(1)}]_{ij}^t (\textbf{U}^\top \textbf{U})_{ii}\left( \textbf{F}_{(1)}(\textbf{W}\otimes \textbf{V})^\top (\textbf{W}\otimes \textbf{V})\textbf{F}_{(1)}^\top \right) _{jj}. \end{aligned}$$

Since

$$\begin{aligned}{} & {} \left[ \textbf{U}^\top \textbf{U}\textbf{A}_{(1)}\textbf{F}_{(1)}(\textbf{W}\otimes \textbf{V})^\top (\textbf{W}\otimes \textbf{V})\textbf{F}_{(1)}^\top \right] _{ij}\\{} & {} \quad =\sum _{i_2=1}^{r_1}\sum _{i_3=1}^{r^2}(\textbf{U}^\top \textbf{U})_{ii_2}[\textbf{A}_{(1)}]_{i_2i_3}[\textbf{F}_{(1)}(\textbf{W}\otimes \textbf{V})^\top (\textbf{W}\otimes \textbf{V})\textbf{F}_{(1)}^\top ]_{i_3 j}\\{} & {} \quad =\sum _{i_2=1,i_2\ne i}^{r_1}\sum _{i_3=1,i_3\ne j}^{r^2}(\textbf{U}^\top \textbf{U})_{ii_2}[\textbf{A}_{(1)}]_{i_2i_3}[\textbf{F}_{(1)}(\textbf{W}\otimes \textbf{V})^\top (\textbf{W}\otimes \textbf{V})\textbf{F}_{(1)}^\top ]_{i_3 j}\\{} & {} \qquad +[\textbf{A}_{(1)}]_{ij}^t (\textbf{U}^\top \textbf{U})_{ii}\left( \textbf{F}_{(1)}(\textbf{W}\otimes \textbf{V})^\top (\textbf{W}\otimes \textbf{V})\textbf{F}_{(1)}^\top \right) _{jj} \\{} & {} \quad \ge [\textbf{A}_{(1)}]_{ij}^t (\textbf{U}^\top \textbf{U})_{ii}\left( \textbf{F}_{(1)}(\textbf{W}\otimes \textbf{V})^\top (\textbf{W}\otimes \textbf{V})\textbf{F}_{(1)}^\top \right) _{jj}, \end{aligned}$$

which implies (23) holds, then $\mathbb {G}(x,[\textbf{A}_{(1)}]_{ij}^t)\ge \mathbb {F}_{ij}(x)$ is satisfied. This completes the proof. $\square$

#### Theorem 1

The objective function of the HNTriD model (7) is non-increasing under the updating rule $\textbf{A}_{(1)}$ represented as (12).

#### Proof

Replacing the auxiliary function $\mathbb {G}(x,x^t)$ of (20) with (21) yields

$$\begin{aligned}{}[\textbf{A}_{(1)}]_{ij}^{t+1}=\arg \min \limits _x \mathbb {G}(x,[\textbf{A}_{(1)}]_{ij}^t). \end{aligned}$$

According to

$$\begin{aligned}{} & {} \frac{\partial \mathbb {G}(x,[\textbf{A}_{(1)}]_{ij}^t)}{\partial x}\\{} & {} \quad =\mathbb {F}_{ij}^\prime ([\textbf{A}_{(1)}]_{ij}^t) +\frac{\left[ \textbf{U}^\top \textbf{U}\textbf{A}_{(1)}\textbf{F}_{(1)}(\textbf{W}\otimes \textbf{V})^\top (\textbf{W}\otimes \textbf{V})\textbf{F}_{(1)}^\top \right] _{ij}}{[\textbf{A}_{(1)}]_{ij}^t}(x-[\textbf{A}_{(1)}]_{ij}^t)=0, \end{aligned}$$

we have

24 $$\begin{aligned} {[}\textbf{A}_{(1)}]_{ij}^{t+1}=[\textbf{A}_{(1)}]_{ij}^t\frac{\left[ \textbf{U}^\top \textbf{X}_{(1)}(\textbf{W}\otimes \textbf{V})\textbf{F}_{(1)}^\top \right] _{ij}}{\left[ \textbf{U}^\top \textbf{U}\textbf{A}_{(1)}\textbf{F}_{(1)}(\textbf{W}\otimes \textbf{V})^\top (\textbf{W}\otimes \textbf{V})\textbf{F}_{(1)}^\top \right] _{ij}}. \end{aligned}$$

Then we can see that (24) agrees with (12), and the Lemma 4 guarantees that (21) is an auxiliary function of $\mathbb {F}_{ij}$. Based on this, in conjunction with Lemma 3, we can get that $f_{HNTriD}$ is non-increasing under the update rule of (12). The proof is then finished. $\square$

We are going to state that the update for $\textbf{W}$ expressed as (17) is equal to the update (20) with an appropriate auxiliary function. Considering the *i*th row and *j*th column entry $\textbf{W}_{ij}$ in $\textbf{W}$, we use $\hat{\mathbb {F}}_{ij}$ to denote the part of the objective function (7) that is only relevant to $\textbf{W}_{ij}$. The first and second derivatives of $\hat{\mathbb {F}}_{ij}$ are shown below

$$\begin{aligned} \hat{\mathbb {F}}_{ij}^\prime =\left[ \left( \textbf{W}\textbf{C}_{(3)}\textbf{H}_{(3)}(\textbf{V}\otimes \textbf{U})^\top -\textbf{X}_{(3)}\right) (\textbf{V}\otimes \textbf{U})\textbf{H}_{(3)}^\top \mathcal {C}_{(3)}^\top +\alpha \textbf{L}\textbf{W}\right] _{ij} \end{aligned}$$

and

$$\begin{aligned} \hat{\mathbb {F}}_{ij}^{\prime \prime }= \left[ \textbf{C}_{(3)}\textbf{H}_{(3)}(\textbf{V}\otimes \textbf{U})^\top (\textbf{V}\otimes \textbf{U})\textbf{H}_{(3)}^\top \textbf{C}_{(3)}^\top \right] _{jj}+\alpha \textbf{L}_{ii}, \end{aligned}$$

respectively.

#### Lemma 5

The function

25 $$\begin{aligned} \hat{\mathbb {G}}(x,\textbf{W}_{ij}^t)= & {} \hat{\mathbb {F}}_{ij}(\textbf{W}_{ij}^t)+\hat{\mathbb {F}}_{ij}^\prime (\textbf{W}_{ij}^t)(x-\textbf{W}_{ij}^t)\nonumber \\{} & {} +\frac{\left[ \textbf{W}\textbf{C}_{(3)}\textbf{H}_{(3)}(\textbf{V}\otimes \textbf{U})^\top (\textbf{V}\otimes \textbf{U})\textbf{H}_{(3)}^\top \textbf{C}_{(3)}^\top +\alpha \textbf{D}_{v} \textbf{W}\right] _{ij}}{2\textbf{W}_{ij}^t}(x-\textbf{W}_{ij}^t)^2 \end{aligned}$$

is an auxiliary function for $\hat{\mathbb {F}}_{ij}$, which is only relevant to $\textbf{W}_{ij}$.

#### Proof

Since $\hat{\mathbb {G}}(x,x)=\hat{\mathbb {F}}_{ij}(x)$ is obvious, we only need to illustrate that the condition $\hat{\mathbb {G}}(x,x)\ge \hat{\mathbb {F}}_{ij}(x)$ holds. To achieve this, we take into consideration the Taylor series expansion of $\hat{\mathbb {F}}_{ij}(x)$ which can be expressed as follows

26 $$\begin{aligned} \hat{\mathbb {F}}_{ij}(x)=\hat{\mathbb {F}}_{ij}(\textbf{W}_{ij}^t)+\hat{\mathbb {F}}_{ij}^\prime (\textbf{W}_{ij}^t)(x-\textbf{W}_{ij}^t)+\frac{1}{2}\hat{\mathbb {F}}_{ij}^{\prime \prime }(\textbf{W}_{ij}^t)(x-\textbf{W}_{ij}^t)^2. \end{aligned}$$

Combing (25) with (26) we can find that $\hat{\mathbb {G}}(x,\textbf{W}_{ij}^t)\ge \hat{\mathbb {F}}_{ij}(x)$ is equivalent to

$$\begin{aligned} \frac{\left[ \textbf{W}\textbf{C}_{(3)}\textbf{H}_{(3)}(\textbf{V}\otimes \textbf{U})^\top (\textbf{V}\otimes \textbf{U})\textbf{H}_{(3)}^\top \textbf{C}_{(3)}^\top +\alpha \textbf{D}_{v} \textbf{W}\right] _{ij}}{2\textbf{W}_{ij}^t}\ge \frac{1}{2} \hat{\mathbb {F}}_{ij}^{\prime \prime }(\textbf{W}_{ij}^t). \end{aligned}$$

And the above equation can be rewritten as

27 $$\begin{aligned}{} & {} \left[ \textbf{W}\mathcal {C}_{(3)}\textbf{H}_{(3)}(\textbf{V}\otimes \textbf{U})^\top (\textbf{V}\otimes \textbf{U})\textbf{H}_{(3)}^\top \textbf{C}_{(3)}^\top +\alpha \textbf{D}_{v} \textbf{W}\right] _{ij}\nonumber \\{} & {} \quad \ge \textbf{W}_{ij}^t \left[ \textbf{C}_{(3)}\textbf{H}_{(3)}(\textbf{V}\otimes \textbf{U})^\top (\textbf{V}\otimes \textbf{U})\textbf{H}_{(3)}^\top \textbf{C}_{(3)}^\top \right] _{jj}+\alpha \textbf{W}_{ij}^t\textbf{L}_{ii}. \end{aligned}$$

Since

$$\begin{aligned}{} & {} \left[ \textbf{W}\mathcal {C}_{(3)}\textbf{H}_{(3)}(\textbf{V}\otimes \textbf{U})^\top (\textbf{V}\otimes \textbf{U})\textbf{H}_{(3)}^\top \textbf{C}_{(3)}^\top +\alpha \textbf{D}_{v} \textbf{W}\right] _{ij}\\{} & {} \quad =\sum _{k=1}^{r_3} \textbf{W}_{ik}\left[ \textbf{C}_{(3)}\textbf{H}_{(3)}(\textbf{V}\otimes \textbf{U})^\top (\textbf{V}\otimes \textbf{U})\textbf{H}_{(3)}^\top \textbf{C}_{(3)}^\top \right] _{kj}+\alpha (\textbf{D}_{v})_{ii}\textbf{W}_{ij}^t\\{} & {} \quad =\sum _{k=1,k\ne j}^{r_3} \textbf{W}_{ik}\left[ \textbf{C}_{(3)}\textbf{H}_{(3)}(\textbf{V}\otimes \textbf{U})^\top (\textbf{V}\otimes \textbf{U})\textbf{H}_{(3)}^\top \textbf{C}_{(3)}^\top \right] _{kj}\\{} & {} \qquad +\textbf{W}_{ij}^t\left[ \textbf{C}_{(3)}\textbf{H}_{(3)}(\textbf{V}\otimes \textbf{U})^\top (\textbf{V}\otimes \textbf{U})\textbf{H}_{(3)}^\top \textbf{C}_{(3)}^\top \right] _{jj}+\alpha \textbf{L}_{ii}\textbf{W}_{ij}^t+\alpha \bar{\textbf{S}}_{ii}\textbf{W}_{ij}^t\\{} & {} \quad \ge \textbf{W}_{ij}^t \left[ \textbf{C}_{(3)}\textbf{H}_{(3)}(\textbf{V}\otimes \textbf{U})^\top (\textbf{V}\otimes \textbf{U})\textbf{H}_{(3)}^\top \textbf{C}_{(3)}^\top \right] _{jj}+\alpha \textbf{W}_{ij}^t\textbf{L}_{ii}, \end{aligned}$$

which implies (27) holds, and $\hat{\mathbb {G}}(x,\textbf{W}_{ij}^t)\ge \hat{\mathbb {F}}_{ij}(x)$ is satisfied. This completes the proof. $\square$

#### Theorem 2

The objective function of the HNTriD model (7) is non-increasing under the updating rule $\textbf{W}$ represented as (17).

#### Proof

Using (25) to replace the $\mathbb {G}(x,x^t)$ that lies in (20), we obtain

$$\begin{aligned} \textbf{W}_{ij}^{t+1}=\arg \min \limits _x \hat{\mathbb {G}}(x,\textbf{W}_{ij}^t). \end{aligned}$$

According to

$$\begin{aligned}{} & {} \frac{\partial \hat{\mathbb {G}}(x,\textbf{W}_{ij}^t)}{\partial x}\\{} & {} \quad =\hat{\mathbb {F}}_{ij}^\prime (\textbf{W}_{ij}^t) +\frac{\left[ \textbf{W}\textbf{C}_{(3)}\textbf{H}_{(3)}(\textbf{V}\otimes \textbf{U})^\top (\textbf{V}\otimes \textbf{U})\textbf{H}_{(3)}^\top \textbf{C}_{(3)}^\top +\alpha \textbf{D}_{v} \textbf{W}\right] _{ij}}{\textbf{W}_{ij}^t}(x-\textbf{W}_{ij}^t) =0, \end{aligned}$$

we have

28 $$\begin{aligned} \textbf{U}_{ij}^{t+1}=\textbf{U}_{ij}^t\frac{\left[ \textbf{X}_{(3)}(\textbf{V}\otimes \textbf{U})\textbf{H}_{(3)}^\top \textbf{C}_{(3)}^\top +\alpha \bar{\textbf{S}} \textbf{W}\right] _{ij}}{\left[ \textbf{W}\textbf{C}_{(3)}\textbf{H}_{(3)}(\textbf{V}\otimes \textbf{U})^\top (\textbf{V}\otimes \textbf{U})\textbf{H}_{(3)}^\top \textbf{C}_{(3)}^\top +\alpha \textbf{D}_{v} \textbf{W}\right] _{ij}}. \end{aligned}$$

It is worth noting that (28) is consistent with (17). Lemma 5 ensures that (25) is an auxiliary function of $\hat{\mathbb {F}}_{ij}$, which combined with Lemma 3 results in $f_{HNTriD}$ being non-increasing under the update rule (17). This brings the proof to a close. $\square$

Applying the same techniques to parameters $\mathcal {B}, \mathcal {C}, \textbf{U}$, and $\textbf{V}$ to check the convergence of HNTriD. To summarize, we can obtain that $f_{HNTriD}$ is non-increasing under each of the update rules for inner factor tensors and matrices $\mathcal {A}, \mathcal {B}, \mathcal {C}, \textbf{U}, \textbf{V}$, and $\textbf{W}$ while fixing the others. Before imposing our algorithm on real-world datasets for clustering tasks, it is necessary to simplify the calculation formulas of the parameters $\mathcal {A}, \mathcal {B}, \mathcal {C}, \textbf{U}, \textbf{V}$, and $\textbf{W}$, as in the following Remark.

#### Remark 1

From the form of updating rules of $\mathcal {A},\mathcal {B},\mathcal {C},\textbf{U}, \textbf{V}$, and $\textbf{W}$, it is a fact that each update needs to calculate the Kronecker products which requires costly storage resources. To simplify the produce of updating for mentioned parameters, we take advantages of the tensor property of the mode-*n* unfolding. Then, we get

$$\begin{aligned} \textbf{X}_{(1)}(\textbf{W}\otimes \textbf{V})=(\mathcal {X}\times _2 \textbf{V}^\top \times _3 \textbf{W}^\top )_{(1)} \end{aligned}$$

and

$$\begin{aligned} \textbf{F}_{(1)}(\textbf{W}\otimes \textbf{V})^\top (\textbf{W}\otimes \textbf{V})=(\mathcal {F}\times _2 \textbf{V}^\top \textbf{V}\times _3 \textbf{W}^\top \textbf{W})_{(1)}, \end{aligned}$$

which means that (12) and (18) can be transformed as

29 $$\begin{aligned} {[}\textbf{A}_{(1)}]_{ij}\longleftarrow {[}\textbf{A}_{(1)}]_{ij}\frac{\left[ \textbf{U}^\top (\mathcal {X}\times _2 \textbf{V}^\top \times _3 \textbf{W}^\top )_{(1)}\textbf{F}_{(1)}^\top \right] _{ij}}{\left[ \textbf{U}^\top \textbf{U}\textbf{A}_{(1)}(\mathcal {F}\times _2 \textbf{V}^\top \textbf{V}\times _3 \textbf{W}^\top \textbf{W})_{(1)}\textbf{F}_{(1)}^\top \right] _{ij}} \end{aligned}$$

and

30 $$\begin{aligned} \textbf{U}_{ij}\longleftarrow \textbf{U}_{ij}\frac{\left[ (\mathcal {X}\times _2 \textbf{V}^\top \times _3 \textbf{W}^\top )_{(1)}\textbf{F}_{(1)}^\top \textbf{A}_{(1)}^\top \right] _{ij}}{\left[ \textbf{U}\textbf{A}_{(1)}(\mathcal {F}\times _2 \textbf{V}^\top \textbf{V}\times _3 \textbf{W}^\top \textbf{W})_{(1)}\textbf{F}_{(1)}^\top \textbf{A}_{(1)}^\top \right] _{ij}}, \end{aligned}$$

respectively. According to

$$\begin{aligned} \textbf{X}_{(2)}(\textbf{W}\otimes \textbf{U})=(\mathcal {X}\times _1 \textbf{U}^\top \times _3 \textbf{W}^\top )_{(2)} \end{aligned}$$

and

$$\begin{aligned} \textbf{G}_{(2)}(\textbf{W}\otimes \textbf{U})^\top (\textbf{W}\otimes \textbf{U})=(\mathcal {G}\times _1\textbf{U}^\top \textbf{U}\times _3 \textbf{W}^\top \textbf{W})_{(2)}, \end{aligned}$$

(13) and (19) can be calculated as

31 $$\begin{aligned} {[}\textbf{B}_{(2)}]_{ij}\longleftarrow [\textbf{B}_{(2)}]_{ij} \frac{\left[ \textbf{V}^\top (\mathcal {X}\times _1 \textbf{U}^\top \times _3 \textbf{W}^\top )_{(2)}\textbf{G}_{(2)}^\top \right] _{ij}}{\left[ \textbf{V}^\top \textbf{V}\textbf{B}_{(2)}(\mathcal {G}\times _1\textbf{U}^\top \textbf{U}\times _3 \textbf{W}^\top \textbf{W})_{(2)}\textbf{G}_{(2)}^\top \right] _{ij}} \end{aligned}$$

and

32 $$\begin{aligned} \textbf{V}_{ij}\longleftarrow \textbf{V}_{ij} \frac{\left[ (\mathcal {X}\times _1 \textbf{U}^\top \times _3 \textbf{W}^\top )_{(2)}\textbf{G}_{(2)}^\top \textbf{B}_{(2)}^\top \right] _{ij}}{\left[ \textbf{V}\textbf{B}_{(2)}(\mathcal {G}\times _1\textbf{U}^\top \textbf{U}\times _3 \textbf{W}^\top \textbf{W})_{(2)}\textbf{G}_{(2)}^\top \textbf{B}_{(2)}^\top \right] _{ij}}. \end{aligned}$$

Similarly, (14) and (17) can be further rewritten as

33 $$\begin{aligned} {[}\textbf{C}_{(3)}]_{ij}\longleftarrow [\textbf{C}_{(3)}]_{ij} \frac{\left[ \textbf{W}^\top (\mathcal {X}\times _1 \textbf{U}\times _2\textbf{V})_{(3)} \textbf{H}_{(3)}^\top \right] _{ij}}{\left[ \textbf{W}^\top \textbf{W}\textbf{C}_{(3)} (\mathcal {H}\times _1 \textbf{U}^\top \textbf{U}\times _2 \textbf{V}^\top \textbf{V})_{(3)} \textbf{H}_{(3)}^\top \right] _{ij}} \end{aligned}$$

and

34 $$\begin{aligned} \textbf{W}_{ij}\longleftarrow \textbf{W}_{ij}\frac{\left[ (\mathcal {X}\times _1 \textbf{U}\times _2\textbf{V})_{(3)}\textbf{H}_{(3)}^\top \textbf{C}_{(3)}^\top +\alpha \bar{\textbf{S}} \textbf{W}\right] _{ij}}{\left[ \textbf{W}\textbf{C}_{(3)}(\mathcal {H}\times _1 \textbf{U}^\top \textbf{U}\times _2 \textbf{V}^\top \textbf{V})_{(3)}\textbf{H}_{(3)}^\top \textbf{C}_{(3)}^\top +\alpha \textbf{D}_{v} \textbf{W}\right] _{ij}}, \end{aligned}$$

respectively.

Hence, the learning rules for the objective function are obtained via the multiplicative update methods described as above. Specifically, we randomly initialize the tensors and factor matrices $\mathcal {A}$, $\mathcal {B}$, $\mathcal {C}$, $\textbf{U},\textbf{V}$, and $\textbf{W}$, then iterate them by (29), (31), (33), (30), (32), and (34). Each iteration ends when the stopping criterion is met. After completing all iterations, we record the operations of the model and examine the convergence at the end of each iteration. The pseudo-code for HNTriD is given in Algorithm 1.

**Algorithm 1:**
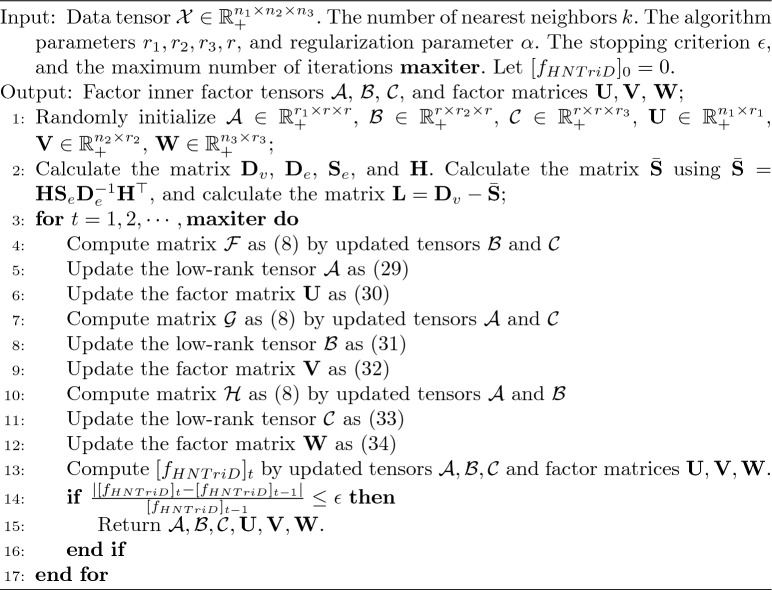
HNTriD algorithm

### Computational complexity analysis

In this subsection, we analyze the computational complexity of the proposed HNTriD model. First, we consider the calculation cost for the tensor-tensor product in (8). In the process of computation tensors $\mathcal {F}, \mathcal {G}$, and $\mathcal {H}$ takes $\mathcal {O}(r^3r_2r_3)$, $\mathcal {O}(r^3r_1r_3)$, and $\mathcal {O}(r^3r_1r_2)$ operations, respectively. It requires $\mathcal {O}(n_1r_1^2+n_2r_2^2+n_3r_3^2)$ operations to calculate symmetric matrices $\textbf{U}^\top \textbf{U}$, $\textbf{V}^\top \textbf{V}$, and $\textbf{W}^\top \textbf{W}$. It takes $\mathcal {O}(n_1n_2n_3r_2+n_1n_3r_2r_3+n_1r^2r_2r_3)$ operations for calculating $(\mathcal {X}\times _2 \textbf{V}^\top \times _3 \textbf{W}^\top )_{(1)}\textbf{F}_{(1)}^\top$. It takes $\mathcal {O}(r^2r_2^2r_3+r^2r_2r_3^2+r^4r_2r_3+r^4r_1+n_1r^2r_1)$ operations to calculate $\textbf{U}\textbf{A}_{(1)}(\mathcal {F}\times _2 \textbf{V}^\top \textbf{V}\times _3 \textbf{W}^\top \textbf{W})_{(1)}\textbf{F}_{(1)}^\top$. Since $(\mathcal {X}\times _2 \textbf{V}^\top \times _3 \textbf{W}^\top )_{(1)}\textbf{F}_{(1)}^\top$ and $\textbf{U}\textbf{A}_{(1)}(\mathcal {F}\times _2 \textbf{V}^\top \textbf{V}\times _3 \textbf{W}^\top \textbf{W})_{(1)}\textbf{F}_{(1)}^\top$ are available, the computational cost for each term, including $\textbf{U}^\top (\mathcal {X}\times _2 \textbf{V}^\top \times _3 \textbf{W}^\top )_{(1)}\textbf{F}_{(1)}^\top$, $\textbf{U}^\top \textbf{U}\textbf{A}_{(1)}(\mathcal {F}\times _2 \textbf{V}^\top \textbf{V}\times _3 \textbf{W}^\top \textbf{W})_{(1)}\textbf{F}_{(1)}^\top$, $(\mathcal {X}\times _2 \textbf{V}^\top \times _3 \textbf{W}^\top )_{(1)}\textbf{F}_{(1)}^\top \textbf{A}_{(1)}^\top$, and $\textbf{U}\textbf{A}_{(1)}(\mathcal {F}\times _2 \textbf{V}^\top \textbf{V}\times _3 \textbf{W}^\top \textbf{W})_{(1)}\textbf{F}_{(1)}^\top \textbf{A}_{(1)}^\top$, is equal to $\mathcal {O}(n_1r^2r_1)$. Then, the cost of computing the update rules of $\mathcal {A}$ in (29) is about $\mathcal {O}(r^2r_1)$. Assume that integers $r_1, r_2, r_3$, and *r* are of the same order of magnitude and they are much smaller than $n_1,n_2$, and $n_3$. We claim that the total computational cost of computing the update rule of $\mathcal {A}$ in (29) and $\textbf{U}$ in (30) is approximately

$$\begin{aligned} \mathcal {O}(n_1n_2n_3r+n_1n_3r^2+n_1r^4+r^6) \end{aligned}$$

Similarly, the total computational cost of computing updating rules for $\mathcal {B}$ in (31) and $\textbf{V}$ in (32) is about

$$\begin{aligned} \mathcal {O}(n_1n_2n_3r+n_2n_3r^2+n_2r^4+r^6). \end{aligned}$$

The total computational cost of updating the rules for $\mathcal {C}$ in (33) and $\textbf{W}$ in (34) is approximately

$$\begin{aligned} \mathcal {O}(n_1n_2n_3r+n_1n_2r^2+n_3r^4+r^6). \end{aligned}$$

Therefore, we can get the total calculation cost of the HNTriD algorithm approximately as

$$\begin{aligned} \mathcal {O}(n_1n_2n_3r+(n_1n_3+n_2n_3+n_1n_2)r^2+(n_1+n_2+n_3)r^4+r^6). \end{aligned}$$

## Experiments

To check the validation of our proposed HNTriD algorithm for clustering data with dimensionality reduction, we run experiments on six popular datasets and compare the results of (7) with that of the related state-of-the-art methods, including NMF^42^, GNMF^18^, HNMF^29^, HSNMF^31^, SHNMF^43^, HGNTR^33^, LraHGNTR^33^, HyperNTF^32^, and TriD^17^. All the simulations will be performed on a desktop computer equipped with an Intel (R) Core (TM) i5-10400F CPU at 2.90 GHz and 16 GB of memory, running MATLAB 2015a in Windows 10.

### Datasets

The clustering performance is evaluated on six widely used datasets, including COIL20, GEORGIA, MNIST, ORL, PIE, and USPS. The general statistical information of the datasets is summarized in Table 2, including the samples, sizes, and categories that were used in the numerical modeling tests of this paper. A brief overview of the mentioned datasets is presented below.

- COIL20 (https://www.cs.columbia.edu/CAVE/software/softlib/coil-20.php): It is a grayscale image dataset comprised of photographs taken from 20 different individuals, and each person was photographed 72 pieces of images from different angles. After resizing each image to $32\times 32$, we can get a third-order tensor $\mathcal {Y}\in \mathbb {R}_{+}^{32\times 32 \times 1,440}$.
- GEORGIA (http://www.anefian.com/research/face_reco.htm): It is a colored JPG image dataset, every image was drawn from 50 people and each person was photographed 15 pieces of images with cluttered backgrounds. The images used in this paper have been converted to grayscale and resized to $32\times 32$. We can obtain a tensor of third order, which defined as $\mathcal {Y}\in \mathbb {R}_{+}^{32\times 32 \times 750}$.
- MNIST (http://yann.lecun.com/exdb/mnist/): It is a handwritten digit image dataset, and each image is $28\times 28$ in size. More than 60,000 digit images were collected in the MNIST dataset range from “0” to “9”. In the numerical tests of this paper, we chose 100 images randomly for each single digit. Thus, the chosen images can be presented as a third-order tensor $\mathcal {Y}\in \mathbb {R}_{+}^{28\times 28\times 1,000}$.
- ORL (https://github.com/saeid436/Face-Recognition-MLP/tree/main/ORL): It is a dataset that includes 400 grayscale face images of 40 different people collected from different facial expressions, various facial details, and varying lighting, and each image is in size of $112\times 92$. A third-order tensor can be defined as $\mathcal {Y}\in \mathbb {R}_{+}^{112\times 92 \times 400}$.
- PIE (http://www.ri.cmu.edu/projects/project_418.html): It is a dataset containing over 40,000 facial images collected from 68 different individuals. These images were taken in a variety of poses, lighting conditions, and expressions. We randomly selected 53 people with 22 different facial images for our numerical tests. We converted them to gray-level and resize them to $32\times 32$. Then the selected images can be expressed as a third-order tensor $\mathcal {Y}\in \mathbb {R}_{+}^{32\times 32 \times 1,166}$.
- USPS (https://www.csie.ntu.edu.tw/cjlin/libsvmtools/datasets/multiclass.html#usps): It is a dataset that includes 11,000 grayscale handwritten digits (from “0” to “9”) that are $16 \times 16$ in size. In the simulation tests of this paper, we chose 100 images at random for each digit. On this basis, we can build a third-order tensor $\mathcal {Y}\in \mathbb {R}_{+}^{16\times 16 \times 1,000}$.

**Table 2:** Descriptions of the relevant six datasets used in this paper.

Item	Dataset					
	COIL20	GEORGIA	MNIST	ORL	PIE	USPS
Sample	1,440	750	1,000	400	1,166	1,000
Size	$32\times 32$	$32\times 32$	$28\times 28$	$112\times 92$	$32\times 32$	$16\times 16$
Category	20	50	10	40	53	10

###  Evaluation metrics

Clustering analysis groups samples only according to the sample data itself and its aim is to group different objects into different groups according to the controlled conditions. The way to evaluate the efficiency of the clustering methods is that objects within groups are similar to each other, while objects differ from group to group. The greater the similarity within the group, the greater the difference between the groups, the better the clustering effect. As we know, the ACC, NMI, and PUR are widely used assessment criteria^44,45^ of clustering algorithm. The accuracy (ACC) can be defined as

$$\begin{aligned} \text {ACC}(\bar{x_i},x_i)=\frac{1}{n}\sum _{1}^{n}\delta (\bar{x_i},map(x_i)), \end{aligned}$$

where *n* is the number of samples in datasets, $\bar{x_i}$ and $x_i$ denote the cluster sample and the original sample, respectively. The symbol $map(\cdot )$ indicates the matchup relationship mapping function, which is responsible for matching the cluster samples and original samples. The symbol $\delta (\cdot ,\cdot )$ is the delta function shown as follows

$$\begin{aligned} \delta (\bar{x_i},map(x_i))=\left\{ \begin{array}{cl} 1, &{}\text {if }x_i\text { is mapped into }\bar{x_i},\\ 0, &{}\text {otherwise}.\\ \end{array}\right. \end{aligned}$$

In general, the agreement between two clusters can be measured with the mutual information ($\text {MI}$), which is widely used in clustering applications. Given two discrete random variables $\bar{X}$ and *X* which stand for the cluster label sets and true label sets, $\bar{x}$ and *x* are selected arbitrarily from $\bar{X}$ and *X*, respectively. Then, the $\text {MI}$ can be measured by

$$\begin{aligned} \text {MI}(\bar{X},X)=\sum _{x\in X}\sum _{\bar{x}\in \bar{X}}p(\bar{x},x)\log \bigg (\dfrac{p(\bar{x},x)}{p(\bar{x})p(x)}\bigg ), \end{aligned}$$

where $p(\bar{x})$ and *p*(*x*) are the edge probability distribution function which denote the probabilities of the samples. The $p(\bar{x},x)$ denotes the joint probability distribution function of $\bar{X}$ and *X* which means that the object belongs to category $\bar{X}$ and category *X* at the same time. To force the score to have an upper bound, we take the $\text {NMI}$ as one of the evaluation criterion, and the definition is

$$\begin{aligned} \text {NMI}(\bar{X},X)=\frac{\text {MI}(\bar{X},X)}{max(\text {T}(\bar{X}),\text {T}(X))}, \end{aligned}$$

where $\text {T}(\bar{X})$ and $\text {T}(X)$ are the entropy of the cluster label set $\bar{X}$ and the entropy of the true label set *X*. In this way, the score ranges of $\text {NMI}(\bar{X},X)$ is from 0 to 1.

The purity ($\text {PUR}$) of a clustering algorithm is a simple assessment format which only have to calculate the proportion of the correct clustering to the total. In other words, the $\text {PUR}$ is to scale the degree of correctness of measurement, the $\text {PUR}$ score of a cluster is observed by a weighted sum of the $\text {PUR}$ values of the respective clusters, which is denoted by

$$\begin{aligned} \text {PUR}(\bar{X},X)=\dfrac{1}{n}\sum _{j=0}^{k}\max _{i}|\bar{x}_j\cap x_i|, \end{aligned}$$

where $\bar{X}=(\bar{x}_1,\bar{x}_2,\ldots ,\bar{x}_k)$ is the cluster category set, the $\bar{x}_i$ denotes the *i*th cluster set. $X=(x_1,x_2,\ldots ,x_k)$ is the original datasets that need to be clustered, $x_i$ represents the *i*th original object. The total number of the objects is *n* that need to be clustered and the function $|\cdot |$ denotes the cardinality of a set.

### Algorithms for comparison

To ensure the clustering performance, we compare the proposed HNTriD model with the following state-of-the-art clustering algorithms.

- NMF^42^: It incorporates nonnegative constraint into two factor matrices decomposed from the original matrix.
- GNMF^18^: It imposes the graph constraint to the coefficient matrix of the NMF method.
- HNMF^29^: It incorporates the hypergraph constraint into the coefficient matrix of the NMF method.
- HSNMF^30^: It imposes the hypergraph constraint on the coefficient matrix based on the $L_{1/2}$-NMF method.
- SHNMF^31^: It takes the sparse hypergraph as a regularization and adds it to the NMF framework.
- HGNTR^33^: It includes the hypergraph constraint on the last TR core tensor and a nonnegative constraint on TR factor tensors.
- LraHGNTR^33^: It is the low-rank approximation of HGNTR.
- HyperNTF^32^: It imposes a hypergraph constraint on the last factor matrix of the CP model and limits all factor matrices to be nonnegative.
- TriD^17^: It is a bilevel form of the triple decomposition of a third-order tensor.

###  Parameters selection

To achieve the best performance, some critical parameters in the experimental simulations needed to be adjusted. In all tests, let $\epsilon =10^{-5}$ and the maximum number of iterations be 1000 unless otherwise specified. We set the regularized term $\alpha$ at the grid of $\{10^{-3}, 10^{-2}, 10^{-1}, 1, 10, 100, 1000\}$, and the *k*-nearest neighbors are chosen from $\{3, 4, 5, 6, 7\}$. The parameters $r_1$ and $r_2$ are integers empirically chosen from $\{3, 4, \dots , 32\}$, and the integer *r* is chosen from $\{2, 3, \dots , 20\}$. Furthermore, we choose the third mode, $r_3$, as the number of categories in the related datasets, as shown in Table 2. In our experiments, we let one of the parameters $r_1,r_2,r,k,\alpha$ varies in the grid given above, and the rest of the parameters were fixed, and the parameters corresponding to the maximum values of NMI in the experiments were recorded. The optimal parameters corresponding to each dataset are given in Table 3.

**Table 3:** List of parameters’ values corresponding to the maximum NMI of HNTriD on six datasets.

Dataset	Optimal parameter					
	$r_1$	$r_2$	$r_3$	*r*	*k*	$\alpha$
COIL20	17	17	20	4	4	1000
GEORGIA	5	5	50	5	3	0.01
MNIST	15	32	10	15	5	10
ORL	11	11	40	11	5	0.01
PIE	8	6	53	19	6	0.01
USPS	3	16	10	2	4	100

**Figure 3:**
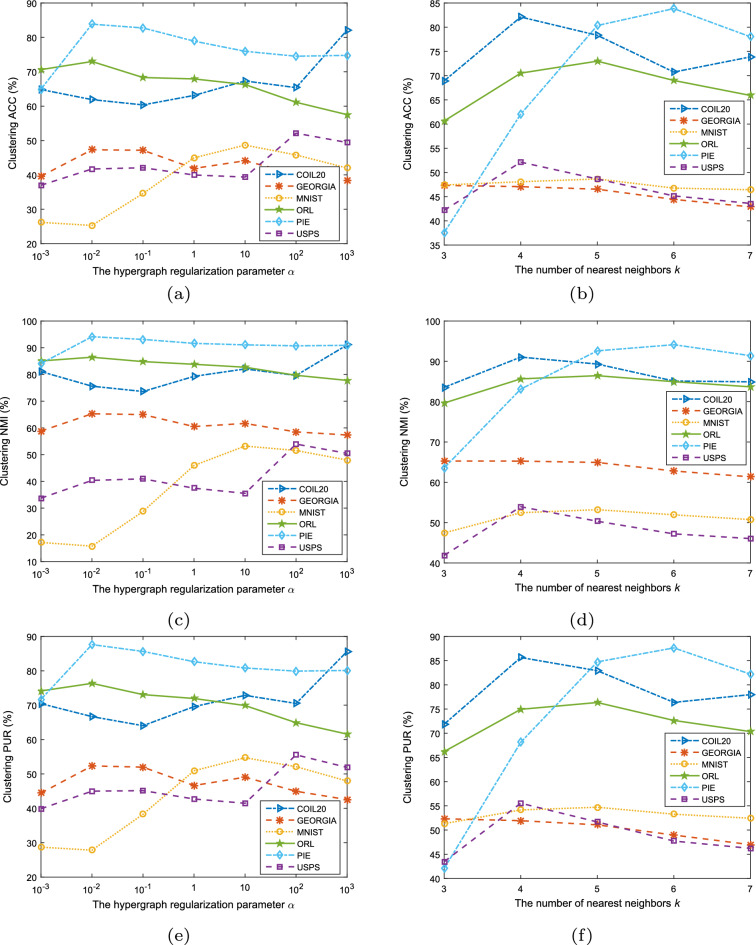
The clustering performance of the HNTriD model varies with different $\alpha$ and the number of nearest neighbors *k*.

In Figure 3, we show the effect of the parameters $\alpha$ and *k* on the three indicators ACC, NMI, and PUR on different datasets. In subplots (a), (c), and (e) of Figure 3, the remaining parameters except $\alpha$ are taken as in Table 3. In subplots (b), (d), and (f) of Figure 3, the remaining parameters except *k* are taken as in Table 3.

From Figure 3, we can conclude that when the parameter $\alpha$ is set to $10^{3}, 10^{-2}, 10, 10^{-2}, 10^{-2}$, and $10^2$, the ACC, NMI, and PUR all perform better in clustering tasks on COIL20, GEORGIA, MNIST, ORL, PIE, and USPS datasets. The parameter *k* is set to 4, 3, 5, 5, 6, and 4, the ACC, NMI, and PUR achieve better results on COIL20, GEORGIA, MNIST, ORL, PIE, and USPS datasets, respectively.

### Convergence study experimentally

**Figure 4:**
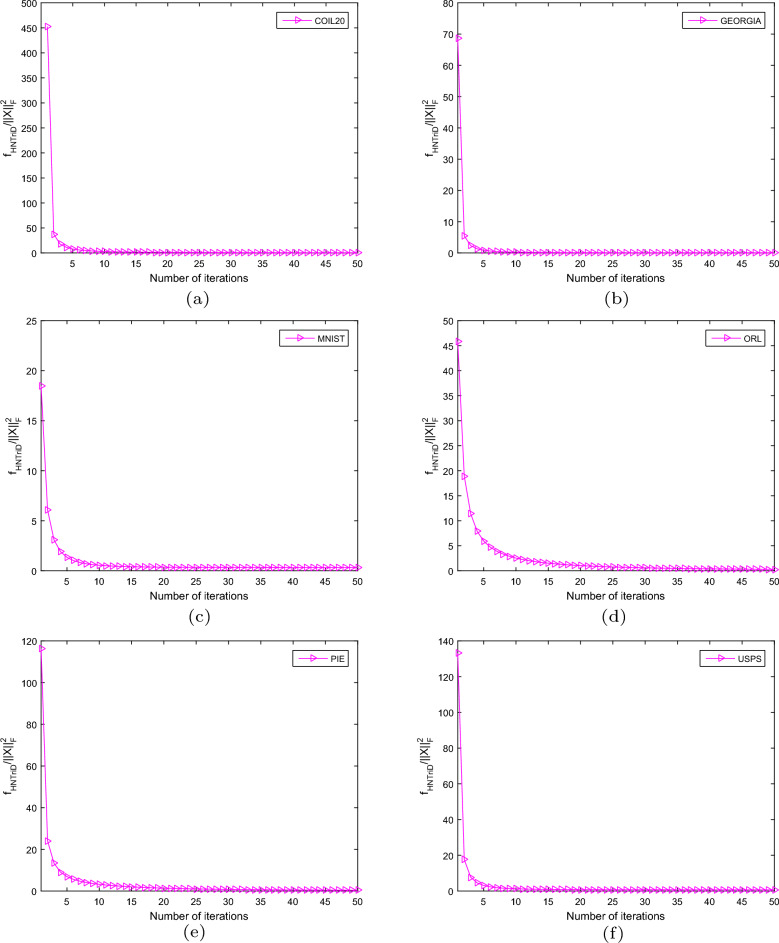
Convergence report of the proposed algorithm on six datasets.

In Section 3.3, we demonstrated that our HNTriD algorithm is non-increasing in theory. Here, we validate it using six HNTriD convergence curves tested from six related datasets, which are shown in Figure 4. There are two important key features that can be identified from Figure 4. First, as the number of iterations increases, the objective function value of HNTriD decreases. Second, the convergence report states that the curve declines rapidly and reaches a relatively stable state within thirty iterations. To summarize, HNTriD experiments show that our method works well on the six datasets mentioned above.

### Experimental comparison

To validate the effectiveness of the proposed HNTriD method, we compare it to some state-of-the-art methods under the assessment criteria ACC, NMI, and PUR. For HNTriD, the parameters are selected as in Table 3. For each method embodied with manifold learning, we set the regularized term $\alpha$ at the grid of $\{10^{-3},10^{-2},10^{-1},1,10,100,1000\}$, and the *k*-nearest neighbors are chosen from {3, 4, 5, 6, 7}. For methods based on TD, such as TriD and HyperNTF, we take the dimension of the third direction of the core tensor to be the class number of the original data. For methods based on tensor ring decomposition, such as HGNTR and LraHGNTR, we take the product of the first order and the third order to be the class number of the original data. The remaining parameters in the comparison algorithm are adjusted on the grid taken by HNTriD. First, we run a number of numerical tests to compare the clustering effect across different datasets. Second, statistical significance comparison is performed on COIL20 and MNIST using the t-test. Third, we present 2-D visualizations of different methods for clustering results on the COIL20 dataset and then complete the comparison tests by means of the t-SNE technique^46^. Finally, we compare the amount of time they took to finish clustering tasks on six related real-world datasets.

#### Numerical comparison results

All experiments are run on the same sub-raw datasets, which are chosen at random from the corresponding database. Each experimental result is obtained only after the process has been repeated 100 times. The numerical tests, in particular, are performed in two steps. The first step is to choose a group of objects at random from the raw data and then decompose them into corresponding sub-raw data based on the parametric form of the model. To ensure that the experimental results are as accurate as possible to the real-world data clustering situation. We repeat the first step 10 times to obtain 10 groups of sub-raw data. In the second step, we use the K-means method to compute the evaluation index value for each group of sub-raw data. As before, we repeat the second step 10 times to obtain 10 evaluation values for each group of sub-raw data. Throughout the experiment, we can receive 100 evaluation index values and calculate the average value as the performance result for each method. Finally, we report the average performance in Tables 4, 5, and 6. Simultaneously, we record the time spent by each method performing clustering tasks on each dataset, and the results are shown in Figure 6.

**Table 4:** Quantitative clustering (ACC%±std%) of different methods on six datasets.

Method	Dataset					
	COIL20	GEORGIA	MNIST	ORL	PIE	USPS
NMF	57.51±4.84	40.52±1.90	48.42±3.72	66.34±3.72	65.62±3.46	41.62±3.18
GNMF	69.32±3.45	41.54±2.10	${\textbf {49.35}}\pm {\textbf {5.78}}$	68.29±3.61	64.84±4.07	44.64±3.35
HNMF	75.13±3.11	41.88±1.69	46.33±5.96	68.61±3.05	64.33±3.45	44.84±3.69
HSNMF	74.59±3.01	38.19±1.83	45.29±5.74	41.28±1.65	$\underline{76.65\pm 1.96}$	40.73±2.52
SHNMF	58.03±4.88	40,52±1.84	48.25±3.59	66.67±3.66	65.63±4.08	41.39±2.43
HGNTR	75.97±3.24	35.79±2.35	44.90±6.42	65.91±3.59	58.90±4.51	50.65±3.21
LraHGNTR	75.61±3.07	$\underline{43.61\pm 1.53}$	43.86±6.27	64.35±3.17	46.32±3.51	47.56±2.99
HyperNTF	$\underline{77.15\pm 4.04}$	41.86±1.51	45.41±4.40	$\underline{71.78\pm 2.23}$	52.34±2.74	${\textbf {52.66}}\pm {\textbf {4.52}}$
TriD	50.57±4.18	31.50±1.53	43.62±4.08	54.30±3.10	74.54±4.25	50.96±5.15
HNTriD	${\textbf {82.11}}\pm {\textbf {2.53}}$	${\textbf {47.32}}\pm {\textbf {2.06}}$	$\underline{48.66\pm 6.14}$	${\textbf {72.99}}\pm {\textbf {2.64}}$	${\textbf {83.85}}\pm {\textbf {3.27}}$	$\underline{52.13\pm 4.08}$

Significant values are bold.

**Table 5:** Quantitative clustering (NMI%±std%) of different methods on six datasets.

Method	Dataset					
	COIL20	GEORGIA	MNIST	ORL	PIE	USPS
NMF	70.82±2.06	60.03±1.13	45.71±2.34	82.77±1.79	85.33±1.39	38.08±2.30
GNMF	82.92±2.58	60.94±1.19	$\underline{52.02\pm 4.58}$	84.57±1.62	84.75±1.80	46.08±3.56
HNMF	87.81±1.52	61.32±1.02	49.92±4.55	84.98±1.52	84.69±1.45	46.18±2.97
HSNMF	87.65±1.60	57.74±1.04	49.26±4.52	61.52±1.09	90.56±0.67	37.08±1.69
SHNMF	72.40±2.37	60.02±1.11	45.66±2.48	83.04±1.82	85.19±1.80	38.30±2.27
HGNTR	88.24±1.43	56.07±1.67	49.42±0.58	82.53±1.64	82.81±1.81	52.33±2.30
LraHGNTR	88.15±1.29	$\underline{62.47\pm 0.91}$	49.57±4.82	82.18±1.51	75.84±2.23	50.16±2.19
HyperNTF	$\underline{88.71\pm 7.12}$	60.78±0.95	48.40±4.18	$\underline{85.27\pm 0.87}$	79.19±1.24	${\textbf {54.07}}\pm {\textbf {2.49}}$
TriD	66.60±2.42	52.10±1.24	38.35±3.39	73.84±2.13	$\underline{90.98\pm 1.69}$	45.82±4.31
HNTriD	${\textbf {91.04}}\pm {\textbf {0.83}}$	${\textbf {65.29}}\pm {\textbf {1.35}}$	${\textbf {53.22}}\pm {\textbf {4.23}}$	${\textbf {86.44}}\pm {\textbf {1.22}}$	${\textbf {94.12}}\pm {\textbf {1.05}}$	$\underline{53.95\pm 2.42}$

Significant values are bold.

**Table 6:** Quantitative clustering (PUR%±std%) of different methods on six datasets.

method	Dataset					
	COIL20	GEORGIA	MNIST	ORL	PIE	USPS
NMF	60.33±3.91	43.27±1.69	52.60±3.33	71.11±2.83	72.09±2.51	43.83±2.63
GNMF	75.21±2.83	44.41±1.80	${\textbf {55.10}}\pm {\textbf {5.29}}$	73.25±2.76	71.31±3.05	47.34±3.17
HNMF	80.52±2.29	44.71±1.55	52.10±5.03	73.69±2.34	70.83±2.56	47.39±2.89
HSNMF	80.20±2.31	40.75±1.65	51.25±4.71	44.02±1.94	80.48±1.53	43.32±2.29
SHNMF	61.12±4.06	43.46±1.70	52.62±3.33	71.48±3.11	71.96±2.96	43.33±2.21
HGNTR	81.19±2.25	38.77±2.15	50.50±6.14	69.98±3.04	64.40±3.63	53.60±2.66
LraHGNTR	81.06±2.05	$\underline{46.71\pm 1.41}$	50.00±5.75	68.67±2.59	51.16±3.41	50.18±2.62
HyperNTF	$\underline{81.80\pm 3.30}$	45.90±1.20	51.20±4.15	$\underline{74.84\pm 1.70}$	59.16±1.99	${\textbf {56,73}}\pm {\textbf {3.54}}$
TriD	54.16±3.40	34.25±1.51	47.27±3.96	59.55±2.73	$\underline{80.64\pm 3.17}$	52.70±4.97
HNTriD	${\textbf {85.65}}\pm {\textbf {1.64}}$	${\textbf {52.33}}\pm {\textbf {1.62}}$	$\underline{54,72\pm 5.32}$	${\textbf {76.36}}\pm {\textbf {2.00}}$	${\textbf {87.63}}\pm {\textbf {2.35}}$	$\underline{55.55\pm 3.55}$

Significant values are bold.

Tables 4, 5, and 6, present experimental results demonstrating correlation algorithm clustering performance on six datasets. The advanced clustering method can be found in the tables under the quantitative clustering rules. For ease of observation, we highlight the best data in bold and the second ones in a slight underline. The experimental results presented above lead us to the following conclusion: (i) In terms of clustering performance, as measured by ACC, NMI, and PUR, our proposed method outperforms others in the majority of cases, and our experimental results are second-best, if not the best. (ii) The best experimental results in the mass are located in tensor-based methods. Because tensor methods consider more information from the raw data. (iii) The HNTriD method outperforms other tensor-based decomposition methods in most cases. Because it inherits the previous algorithm’s excellent characteristics, including TriD, and preserves the data’s multi-linear structure. Experiments show that the proposed HNTriD algorithm performs well in clustering tasks.

#### Statistical significance comparison

A t-test is a statistical technique used to determine if there is a significant difference between two groups of data. It functions as an important tool in hypothesis testing and aids researchers in determining whether two groups are genuinely distinct^47,48^. Subsequently, we examine the statistical significance of the disparity between HNTriD and some typical approaches using t-test. Similar to^49^, we take the significance level of $p < 0.05$ in the t-test to draw the difference. If our approach outperforms a compared method in a comparison test and the difference is statistically significant (t-test, $p < 0.05$), we record it as significant better or worse for one time. If the difference between our approach and a compared method is not statistically significant, then we say that they are comparable. We use (*a*, *b*, *c*) to display comparison results. Three integers inside the brackets respectively correspond to the number of times that the performance of our method is significantly better than, comparable to, significantly worse than a related method. We compare 10 algorithms (including HNTriD) on both the COIL20 and MNIST datasets. In each comparison, we run all the compared algorithms 10 times, and we repeat each group of comparison experiments 10 times. Specifically, the statistical test results are presented in Tables 7 and 8.

**Table 7:** The t-test comparison results of different methods on COIL20.

	Metrics	NMF	GNMF	HNMF	HSNMF	SHNMF	HGNTR	LraHGNTR	HyperNTF	TriD
HNTriD	ACC	(10,0,0)	(10,0,0)	(10,0,0)	(10,0,0)	(10,0,0)	(10,0,0)	(10,0,0)	(10,0,0)	(9,1,0)
	NMI	(10,0,0)	(10,0,0)	(10,0,0)	(10,0,0)	(10,0,0)	(10,0,0)	(10,0,0)	(10,0,0)	(10,0,0)
	PUR	(10,0,0)	(10,0,0)	(10,0,0)	(10,0,0)	(10,0,0)	(10,0,0)	(10,0,0)	(10,0,0)	(10,0,0)
	overall	(30,0,0)	(30,0,0)	(30,0,0)	(30,0,0)	(30,0,0)	(30,0,0)	(30,0,0)	(30,0,0)	(29,1,0)

**Table 8:** The t-test comparison results of different methods on MNIST.

	Metrics	NMF	GNMF	HNMF	HSNMF	SHNMF	HGNTR	LraHGNTR	HyperNTF	TriD
HNTriD	ACC	(3,5,2)	(3,5,2)	(5,3,2)	(10,0,0)	(2,4,4)	(3,6,1)	(7,2,1)	(7,1,2)	(5,5,0)
	NMI	(9,1,0)	(6,3,1)	(7,2,1)	(10,0,0)	(8,2,0)	(7,2,1)	(9,1,0)	(8,1,1)	(10,0,0)
	PUR	(6,4,0)	(6,3,1)	(6,6,2)	(10,0,0)	(4,4,2)	(3,6,1)	(6,4,0)	(9,0,1)	(9,1,0)
	overall	(18,10,2)	(15,11,4)	(18,7,5)	(30,0,0)	(14,10,6)	(13,14,3)	(22,7,1)	(24,2,4)	(24,6,0)

According to comparison tests show in Table 7, our method is significantly superior to the compared methods on the COIL20 dataset. The experimental results on MNIST demonstrate a clear decline in performance compared to the experimental findings on COIL20. However, from the overview of all metrics’ evaluation, the results still demonstrate a high level of performance when compared to other approaches. Based on the information provided in Tables 7 and 8, it is evident that our method demonstrates significant statistical advancements compared to the listed methods in most cases. The statistical test findings indicate that our method has a significantly bigger advantage over the other compared ones.

#### Visualization on clustering tasks

**Figure 5:**
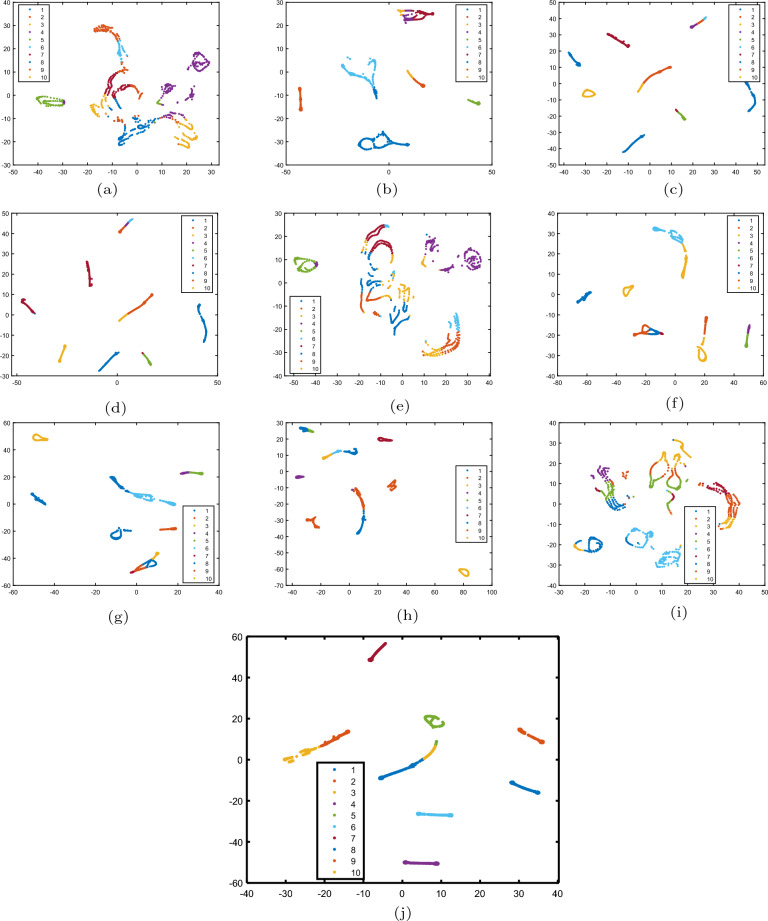
2-D visualizations of the clustering results of several algorithms using t-SNE on the COIL20 dataset. (**a**) NMF. (**b**) GNMF. (**c**) HNMF. (**d**) HSNMF. (**e**) SHNMF.  (**f**) HGNTR. (**g**) LraHGNTR. (**h**) HyperNTF. (**i**) TriD.  (**j**) HNTriD.

In order to visually demonstrate the clustering performance of HNTriD, we present cluster visualizations of several comparable approaches to assess the data learning capability of HNTriD. In this experiment, we choose the COIL20 dataset as a representative example to conduct comparative tests on clustering tasks. We specifically select 10 categories of data for analysis. The data analysis is shown in a two-dimensional space using t-SNE, and the cluster results are displayed in Figure 5 for visual comparison.

Figure 5 demonstrates that the HNTriD method, when applied to the mulitiway dataset, is capable of effectively discerning the differences between data samples. HNTriD outperforms other approaches in visually separating sample clusters in the COIL20 dataset, while some methods fail to completely separate samples from other clusters. This strategy enhances the reliability of the clustering data comparison experiment mentioned above and confirms that the inclusion of HNTriD improves the learning capability of multiway data.

#### Running time comparison

**Figure 6:**
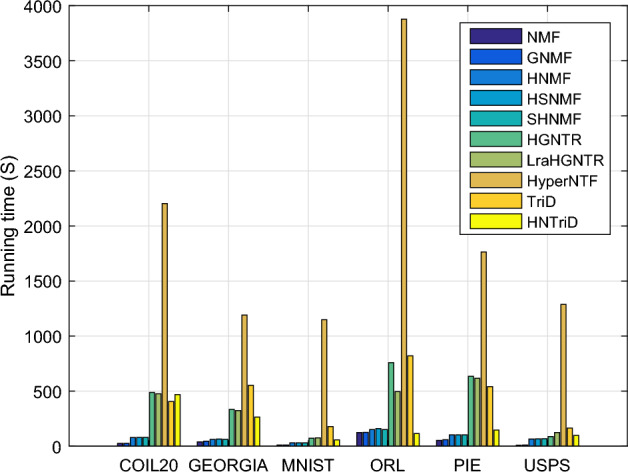
Comparison report of the running time in regard to the different methods on six datasets.

From the previous experimental results (including numerical experiments, statistical significance comparison, and visualization on clustering tasks), the HNTriD model shows better data analysis performance. However, it is important to take into account the time cost when applying mathematical models in real-life situations. This means that if we can improve the efficiency of calculations while preserving the quality of data analysis, the mathematical model will be more effective in practical applications. Based on this background, we figure out the time cost and use Figure 6 to record the running time of clustering tasks for each method on six related datasets. On each dataset, we compare the computational time required by each method to complete the same numerical tests described in Subsection 4.6. Each bar in the Figure 6 represents the total time needed for a method to complete the cluster analysis of a dataset, and different colors represent different algorithms. For example, for each dataset, the time cost of HNTriD is represented in yellow.

We can deduce the following statements from the bar graph: (i) Matrix-based decomposition methods are almost always faster than tensor-based ones. Matrix-based methods have an obvious advantage in terms of running speed for there are few factors that needed to be updated due to their special arithmetic expression. (ii) When compared to general methods, manifold learning ones take longer to complete clustering tasks in most cases. This occurs because manifold learning algorithms require updating more parameters in clustering data. (iii) When compared to matrix-based algorithms, the HNTriD algorithm takes longer to cluster tasks. Given the computational complexity of the algorithm, the experimental results are consistent with our expectations. The increase in computational time is due to the construction of the hypergraph and the depiction of raw data. (iv) Among the tensor-based methods, the HNTriD algorithm’s computation speed does not fall behind while maintaining its superior performance.

## Conclusions

In this paper, the proposed HNTriD method performs well in multiway data learning because it combines the advantages of hypergraph learning and TriD. By constructing hypergraphs, it can reveal the complex structural information of more complex variables hidden among raw data. When combined with the TriD model, it can retain the multi-linear structure of high-order data while mining the potential information within the data and has strong data clustering abilities. Furthermore, we use the multiplicative update method to optimize the proposed HNTriD model, and experiments show that the new algorithm is convergent. The proposed algorithm is applied to six real-world datasets for clustering analysis, including COIL20, GEORGIA, MNIST, ORL, PIE, and USPS, and the data clustering results are compared to those of several existing algorithms. The experimental results demonstrate that the proposed HNTriD method is efficient and saves time in data analysis. In our current work, our hypergraph does not change once it is generated, which may result in a less-than-ideal hypergraph learned in some data with unexpected noise. The solution to this problem, however, is outside the scope of our current work, and we hope to improve it in the future.

## Data Availability

The open datasets used in this manuscript have been linked in related parts, and the datasets generated during the current study are available from the corresponding author on reasonable request.
